# Differential suitability of reactive oxygen species and the role of glutathione in regulating paradoxical behavior in gliomas: A mathematical perspective

**DOI:** 10.1371/journal.pone.0235204

**Published:** 2020-06-25

**Authors:** Rupa Bhowmick, Ram Rup Sarkar

**Affiliations:** 1 Chemical Engineering and Process Development Division, CSIR-National Chemical Laboratory, Pune, Maharashtra, India; 2 Academy of Scientific & Innovative Research (AcSIR), Ghaziabad, India; Duke University School of Medicine, UNITED STATES

## Abstract

Manipulative strategies of ROS in cancer are often exhibited as changes in the redox and thiol ratio of the cells. Cellular responses to oxidative insults are generated in response to these changes which are triggered due to the rerouting of the metabolic framework to maintain survival under stress. However, mechanisms of these metabolic re-routing are not clearly understood and remained debatable. In the present work, we have designed a context-based dynamic metabolic model to establish that the coordinated functioning of glutathione peroxidase (*GTHP*), glutathione oxidoreductase (*GTHO*) and NADPH oxidase (*NOX*) is crucial in determining cancerous transformation, specifically in gliomas. Further, we propose that the puzzling duality of ROS (represented by changes in *h*_*2*_*o*_*2*_ in the present model) in exhibiting varying cellular fates can be determined by considering simultaneous changes in *nadph/nadp*^*+*^ and *gsh/gssg* that occur during the reprogramming of metabolic reactions. This will be helpful in determining the pro-apoptotic or anti-apoptotic fate of gliomas and can be useful in designing effective pro-oxidant and/or anti-oxidant therapeutic approaches against gliomas.

## 1. Introduction

Reactive Oxygen Species (ROS) have been implicated in various disease conditions and are considered as a key driving factor in the process of aging and carcinogenesis. ROS is referred to possess a double-edged sword property having both tumor-promoting and a tumor-suppressing function [1]. An intricate balance between ROS and antioxidants and other ROS scavengers is maintained in a normal proliferating cell, which is a prerequisite for maintaining redox balance and proper functioning of the cell. Human cells generally tend to function in a reduced state (e.g. by maintaining high *gsh/gssg* ratio [2] and high *nadph/nadp*^*+*^ ratio [3]). However, exceptions are made when the cells need to maintain a slightly oxidative environment to aid various cellular processes like folding of nascent proteins in the endoplasmic reticulum, activation of gene transcription factors, etc. An increase in intracellular oxidative state induces apoptosis, although too much oxidation helps to evade apoptosis by oxidizing and inactivating the caspase enzymes [4]. Thus the balance between oxidant and antioxidant activity becomes crucial, as a shift might facilitate apoptosis or might also suppress apoptosis rendering therapeutic approaches ineffective.

ROS are implicated as harmful byproducts of cellular metabolism, although they perform additional function as regulators of various intracellular signaling cascades [5–10]. Sequential reduction of oxygen results in the formation of superoxide, hydroxyl radical, hydrogen peroxide (*h*_*2*_*o*_*2*_), hydroxyl ion, etc. Most of these are converted into hydrogen peroxide by the activity of superoxide dismutase. *h*_*2*_*o*_*2*_ is one of the most stable ROS, which can penetrate membranes and quickly reach cellular targets affecting overall cellular functioning [11]. Responses of ROS differ under different experimental conditions; they can promote proliferation [12, 13] or can inhibit it [14] or can also induce apoptosis [15, 16]. These responses are governed by multiple cellular factors, anti-oxidant metabolism being the most crucial one. A constraint, however, is to obtain a holistic understanding of ROS and its regulatory mechanisms due to contextual [1, 17, 18] and experimental complexities [12, 14, 19, 20]. The use of computational approaches has been conducive in dealing with these limitations. Computational models allow observing the concurrent dynamics of many variables considered simultaneously, which are often challenging to assess experimentally [10, 21].

The differential suitability of ROS manipulation in tumor cells is explained by different theories. One of the most prevalent theories is the “Threshold concept for cancer therapy”. According to this theory, as the level of ROS within the cancer cell increases, the ratio of ROS and antioxidants reaches a balance, beyond which any further increase in ROS or decrease in antioxidant activity will lead to cell death or increased sensitivity of tumor cells to cytotoxic treatments [22]. According to an alternate threshold theory, when both tumor and normal cells are exposed to equal intensity of exogenous ROS-producing or stimulating agents, the intracellular ROS levels of tumor cells increase more easily than the normal cells to reach a threshold and to trigger death, due to higher basal level of ROS in tumors [23]. The changes induced during a cancerous transformation are readily reflected as a change in the redox status of the cells mostly triggering ROS production. The applicability and effectiveness of ROS promotion or ROS depletion strategies in cancer therapeutics depend on where the cell is in the sequence of events. Change in the thiol ratio of the cell is another important determinant of the cellular response towards an oxidative insult. Reportedly, changes in the *nadph/nadp*^*+*^ ratio and *gsh/gssg* ratio have been considered important in gaining a perspective towards the cellular response to such insults. *nadph/nadp*^*+*^ ratio is indicative of the reducing potential of the cell, which is required to be maintained high to keep the overall redox pool at a significantly reduced state [24, 25]. Changes in *gsh/gssg* ratio might induce the initiation of the induction phase of apoptosis [26, 27]. A decrease in *gsh/gssg* ratio can induce apoptosis by causing Bcl-2 loss and activating caspase enzymes whereas an increase in the ratio may have an effect otherwise [28].

Thiol and redox ratios represent cumulative results of multiple changes in the metabolism and hence are considered as indicators of various diseased states. Although, the effect of ROS manipulation on these ratios and the metabolic re-routing that helps the cells to adapt to stressful conditions affect the redox and thiol status of the cell are not clearly understood. Another arguable topic of discussion is whether the redox and thiol statuses of the cell have any role in determining cellular fate during oxidative stress. The well-orchestrated metabolic processes related to these changes are re-routed, although the changes that govern the puzzling duality of ROS are yet not clearly understood. The role of the *gsh-gssg* in determining the paradox is another aspect to be explored. Also, the changes in the metabolic network that affect the effectiveness of pro-oxidant or anti-oxidant approaches during cancer therapeutic design are not well understood. In order to address these questions, we have designed a context-based dynamic metabolic model including the pathways which are known to be involved in regulating the oxidant-antioxidant mechanism.

In the present work, we have demonstrated the effect of redox and thiol status of the cell, and the antioxidants in maintaining ROS levels by considering *h*_*2*_*o*_*2*_ levels in particular in normal glial cells and gliomas. With the help of our simulations, we propose that the effectiveness of a therapeutic strategy depends on the target’s ability to alter the redox states of the cell. We propose a set of sequential changes in metabolic events that determines the transformation from a normal glial to glioma condition that can be used for a pro-oxidative or anti-oxidative therapeutic approach. We have demonstrated these changes keeping in view the changes in *nadph/nadp*^*+*^ and *gsh/gssg* ratios with respect to the *h*_*2*_*o*_*2*_ levels in normal glial and glioma conditions. A holistic understanding of the changes in *h*_*2*_*o*_*2*_ levels along with changes in the redox and thiol statuses of the model provides convincing insight into the paradoxical behavior of ROS in gliomas and its cellular apoptotic fates. Also, understanding the effect of other enzymes that are not directly involved in ROS manipulation but affect the metabolic process by creating a distant regulation in the network can help augment the effect of proposed therapeutic strategies. In the future, this can be ventured to propose an effective design of pro-oxidant or anti-oxidant therapeutic approaches against glioma progression.

## 2. Materials and methods

### 2.1. Model setup

**Fig 1:**
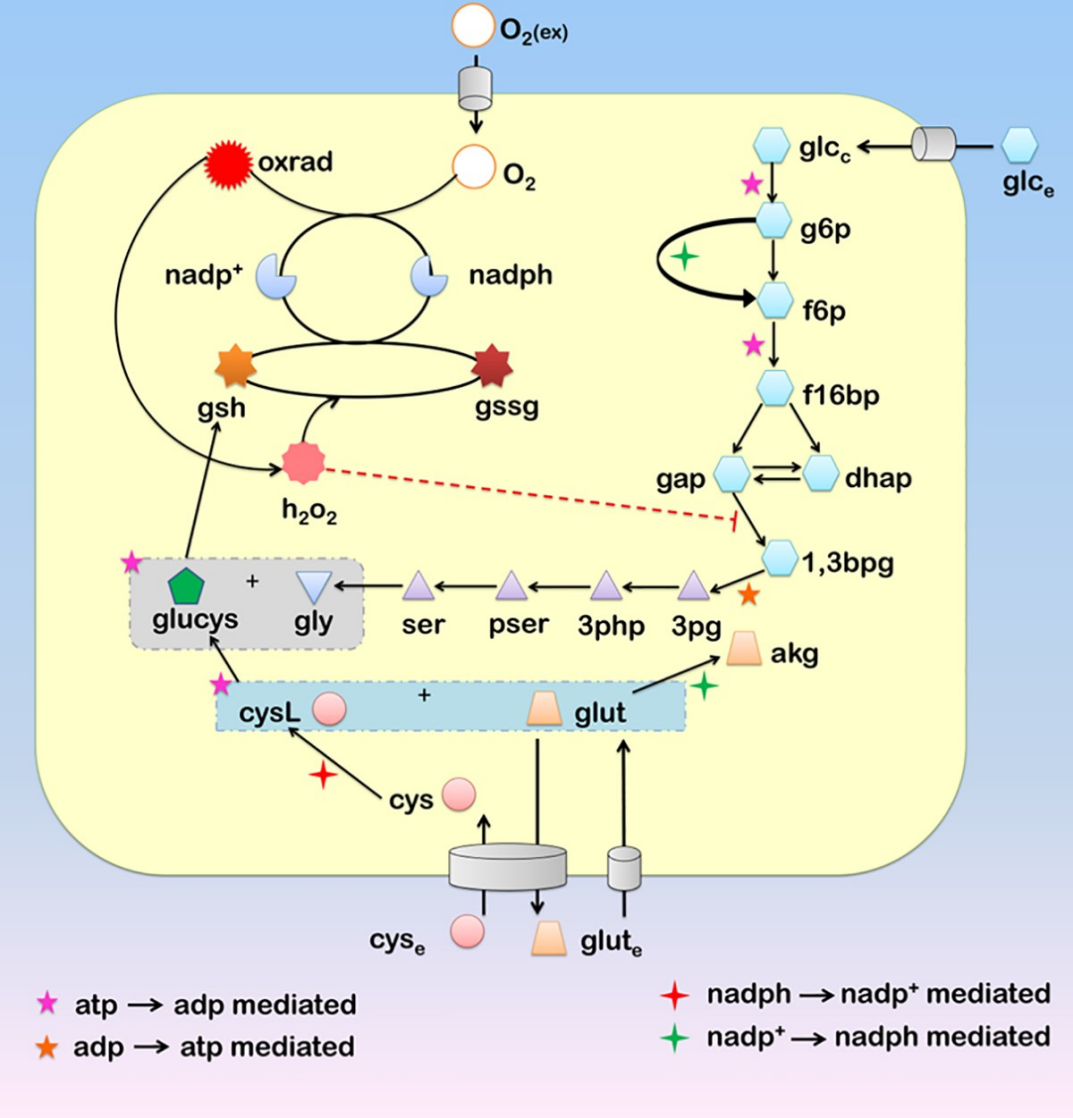
Diagrammatic representation of the metabolites belonging to different pathways directed towards the production of the glutathione along with the ROS generation machinery. The metabolites and the pathways to which they belong have been grouped into five groups viz.-(i) Central Carbon metabolism (*glc*_*e*_, *glc*_*c*_, *g6p*, *f6p*, *f16bp*, *gap*, *dhap*, *1*,*3bpg*, *3pg*, *akg*), (ii) Amino Acid metabolism (*3php*, *pser*, *ser*, *gly*, *cys*_*e*_, *cys*, *cysL*, *glut*_*e*_, *glut*), (iii) Thiol metabolism (*glucys*, *gsh*, *gssg*), (iv) h_2_o_2_ production and metabolism (*O*_*2*_*(ex)*, *O*_*2*_, *oxrad*, *h*_*2*_*o*_*2*_) (v) Redox metabolism (*nadph*, *nadp*^*+*^). Descriptions to all the abbreviated forms have been provided in S1 Text Section 4.

The present model (Fig 1) captures the dynamic changes in the metabolism regulating the interplay of glutathione and ROS (hydrogen peroxide) in normal glial cells and gliomas. The model is biologically motivated from a previous analysis in which a subset of metabolic reactions has been observed to be directed towards *gsh* production in gliomas [29]. A re-routing of the glycolytic, pentose phosphate, glycine-serine, glutamate, and cystine pathway was observed to be directed towards glutathione metabolism from the analysis. The present model has been designed considering these pathways, to understand the dynamics of this re-routing and their effect in determining the role of glutathione during ROS scavenging. ROS which typically show a paradoxical behavior in tumor progression and proliferation have been represented in the model with the inclusion of ROS production machinery. *gsh* is a tri-peptide composed of cystine (*cys*), glycine (*gly*) and glutamate (*glut*), is the prime anti-oxidant involved in ROS scavenging. The model essentially comprises of reactions required for the production of the components of tri-peptide units of *gsh*. A part of glycolytic pathways along with the glycine-serine metabolism has been included which directs the glucose metabolism towards glycine production. Cystine metabolism and a part of the glutamate metabolism have been incorporated to represent the metabolism of these two components into the complex- glutamyl-cysteine (*glucys*). The *gsh-gssg* cycle consisting of glutathione peroxidase (*GTHP*) and glutathione oxidoreductase (*GTHO*) has been included along with the ROS production machinery comprising of NADPH Oxidase (*NOX*) and Superoxide Dismutase (*SOD*). Although these enzymes are present as multiple isoforms, all the isoforms have been considered as one, as their basic mechanism and functioning remains the same. Other ROS scavenging machinery like the peroxiredoxin/thioredoxin systems, catalases, etc., have not been considered in the model for time being, as we intended to focus on the dynamics of glutathione during ROS scavenging and its role in determining the paradox. The scavenging of *h*_*2*_*o*_*2*_ (which has been considered as the ROS in the present model) by other mechanisms has been represented by the parameter *dh*_2_*o*_2_, which is defined as the decay of intracellular hydrogen peroxide in other cellular processes. The model has been limited to two compartments only: extracellular matrix (e) and cytosol (c) and intracellular compartments have not been considered as the availability of compartment-wise parameter remains a limitation and the introduction of intracellular compartments for such large ODE model would make it complex.

### 2.2. Model description

The model consists of 35 metabolite variables involved in 25 reactions required for the production of *gsh* and *h*_*2*_*o*_*2*_ and for scavenging *h*_*2*_*o*_*2*_. All reactions have been provided in supporting information. As per the available information of the kinetics of these reactions, equations have been formulated as forms of uni-uni, bi-bi, bi-uni or ter-bi Michaelis-Menten kinetics. The equations have been written in Cleland Nomenclature. We have considered initial reaction kinetics with first-order reaction rates. All the rate equations, differential equations and parameter description along with the parameter values have been provided in S1 Table of S1 Text.

The general form of the initial kinetics of reaction equations considered in the model is given below:

$$Uni-Uni/Uni-Bi:\frac{V_{m}^{R}.a}{K_{m}^{R}+a}$$

$$Bi-Bi\;mechanism\;(Rapid\;equilibrium/Ordered):\frac{V_{m}^{R}.(a.b)}{\left(k_{i(a)}^{R}.k_{b}^{R}+k_{b}^{R}.a+k_{a}^{R}.b+a.b\right)}$$

$$Partial\;Rapid\;Equilibrium\;Random\;Bi-Bi\;mechanism:\frac{V_{m}^{R}.(a.b)}{\left(k_{i(a)}^{R}.k_{b}^{R}+k_{b}^{R}.a+a.b\right)}$$

$$Ordered\;Ter-Bi:\frac{V_{m}^{R}.(a.b.c)}{\left(k_{i(a)}^{R}.k_{i(b)}^{R}.k_{c}^{R}+k_{i(b)}^{R}.k_{c}^{R}.a+k_{i(a)}^{R}.k_{b}^{R}.c+k_{c}^{R}.a.b+k_{b}^{R}.a.c+k_{a}^{R}.b.c+a.b.c\right)}$$

Where *R* is the reaction, *a*, *b* and *c* represent the substrates, $V_{m}^{R}$ the V_max_ of the reaction, $k_{i}^{R}$ the rate constant for dissociation of enzyme-substrate complex and $k_{a}^{R}/k_{b}^{R}/k_{c}^{R}$ represent the rate constant for the association of a substrate with the enzyme.

The motivation behind considering a larger metabolic network for dynamic analysis comes from the observed metabolic re-routing of these pathways which is directed towards glutathione production. In the present model, our main focus remains to understand the strategies of *h*_*2*_*o*_*2*_ manipulation within cells and its effect on cell proliferation or death. With this model, we also try to understand the effect of glutathione in scavenging *h*_*2*_*o*_*2*_ while considering the changes in the redox ratio depicted by *nadph/nadp*^*+*^. The rationale behind considering the different pathways is specified hereunder.

#### Central carbon metabolism

The glycolytic pathway branches out to various other pathways which are precursors to the nucleotide, amino acid synthesis, and other important biosynthesis pathways. A part of the glycolytic pathway which branches to serine metabolism has been considered which allows the de novo synthesis of the amino acids serine and glycine within the cell. Also, the pentose phosphate pathway has been represented by the inclusion of G6PDH which is a major source of *nadph*.

#### Amino acid metabolism

All reactions belonging to serine and glycine metabolism has been considered in the model. This metabolism supports the generation of glutathione and maintains redox status of the cell. Serine and glycine can be produced *de novo* from glycolytic intermediate 3*pg*, and this part of the metabolism has been considered in the model [30]. Glutamate metabolism has been considered partly. This forms another source of *nadph*, and glutamate supports the production of glutathione and also manages the uptake of cystine through the cystine-glutamate antiporter. Cysteine metabolism has also been considered. The initiation of the metabolism has been considered with the uptake of cystine which is metabolized to cysteine [31]. This reacts with glutamate to form glutamyl-cysteine (*glucys*), the precursor complex which reacts with glycine to produce glutathione [26].

#### Thiol metabolism

The thiol metabolism is represented by the glutathione metabolism itself. Two important enzymes that maintain the glutathione homeostasis i.e., glutathione peroxidase and glutathione oxidoreductase, have been considered [27]. Pertaining to the objective of looking into the glutathione and *h*_*2*_*o*_*2*_ dynamics, other thiol metabolisms like the peroxiredoxins, thioredoxins, and catalase systems have not been considered currently.

#### h_2_o_2_ production and metabolism

The model considers the activity of NADPH oxidase [32] and superoxide dismutase [33] as a part of *h*_*2*_*o*_*2*_ *production*. The formation of oxygen free radicals and their subsequent conversion into *h*_*2*_*o*_*2*_ is catalyzed by NADPH oxidase and superoxide dismutase respectively. The metabolism of *h*_*2*_*o*_*2*_ is linked to thiol metabolism. Other mechanisms through which *h*_*2*_*o*_*2*_ are scavenged is represented by the parameter *dh*_2_*o*_2_, which is defined as the decay of intracellular hydrogen peroxide in other cellular processes.

#### Redox metabolism

*nadph- nadp*^*+*^ has been considered as the prime redox balancer in the model. The homeostasis and metabolism of *nadph- nadp*^*+*^ has been considered in reactions belonging to the earlier pathways where these function as cofactors to catalyze the reactions.

### 2.3. Model simulation

The system of differential equations was simulated using ODE15s in MATLAB 2017a. Calibration of the model was performed upon available experiment data on the change in *gsh* over a period of time in retinal Muller glial cells under normal and amino acid supplemented scenarios [34]. This was used to create the normal glial scenario, to which changes were introduced to create the hypoxic and glioma-like scenario. The basal parameters and initial values to the variables were considered within a biologically feasible range as obtained through literature search and are provided in S2 Table of S1 Text. Few of the parameters and initial values were assumed within a biologically feasible range as had been reported in the literature. A set of 23 parameters remained unknown which were determined using the parameter estimation technique. The model was ensured to reach a stable steady state with the basal parameter states and initial values. Also, the simulations performed using the above parameters indicate that at steady states the intracellular *gsh* concentration along with *nadph/nadp*^*+*^ and *atp/adp* ratios resemble true biological concentrations as reported in the literature.

### 2.4. Parameter estimation

**Fig 2:**
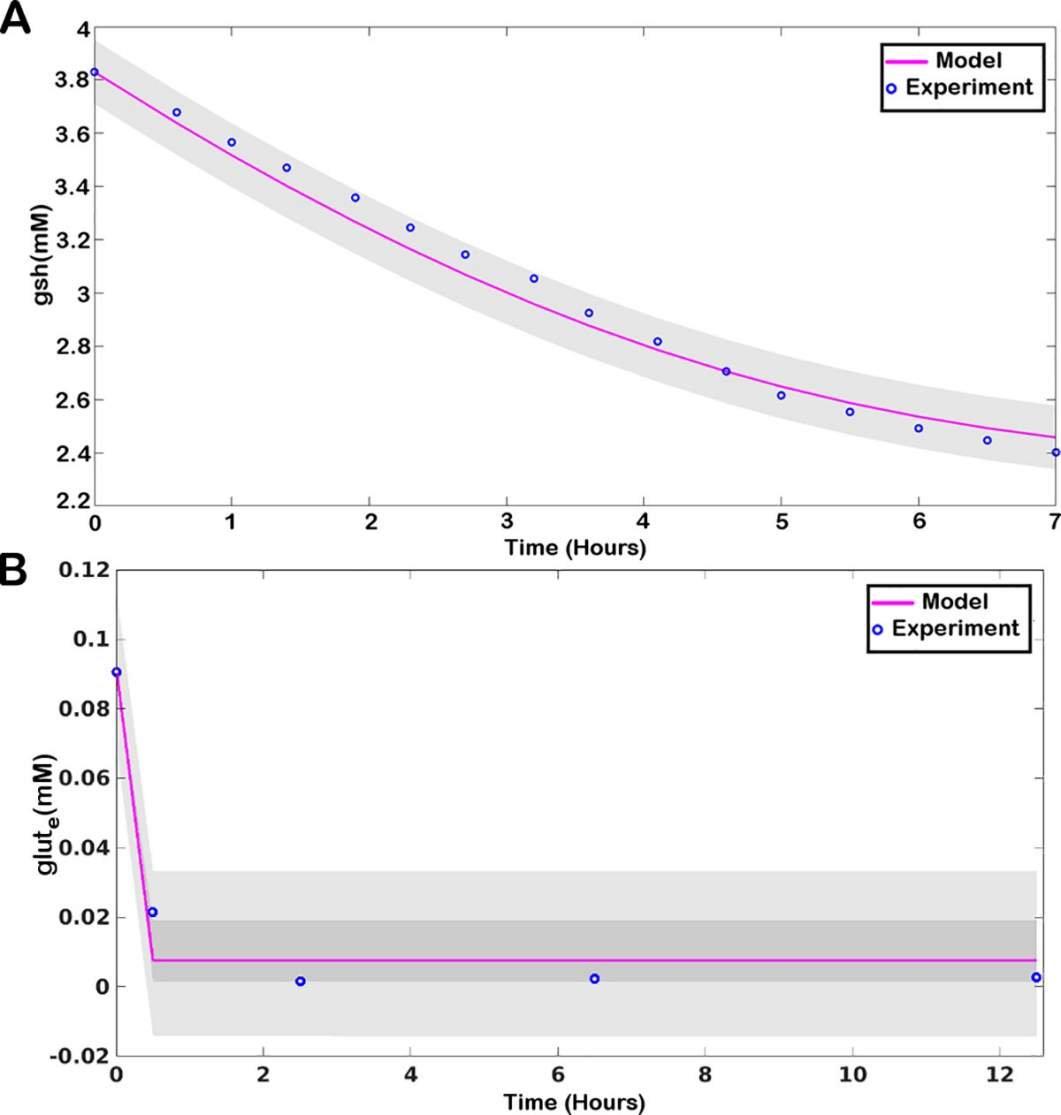
Predictive plot demonstrating of model fitting with experimental data: (A) for reduced glutathione (*gsh*) and (B) for extracellular glutamate (*glut*_*e*_). The blue circles represent the data points obtained from experiments [34, 35] and the pink line represents the result obtained through model simulation.

Parameter estimation of the unknown parameters was performed using Delayed Rejection Adaptive Metropolis (DRAM) algorithm of Markov Chain Monte Carlo (MCMC) Toolbox in MATLAB 2017a. The algorithm generates posterior distribution calibrated using the sample path of the MCMC chain to estimate unknown parameters for a known experimental result. In the present model, parameter estimation was performed using experimental data on the change in *gsh* in retinal Muller glial cells as has been specified in the earlier section (Fig 2A). Distributions plots and trace plots of all the 23 estimated parameters have been provided in S1 Fig of S1 Text. Values for the parameters $V_{m}^{GLUTEX}$ and $k_{m}^{GLUTEX}$ were estimated using an available data on glutamate exchange in astrocytes [35] (Fig 2B). Distributions plots and trace plots of the two estimated parameters have been provided in S2 Fig of S1 Text.

### 2.5. Model Validation with experimental data

**Fig 3:**
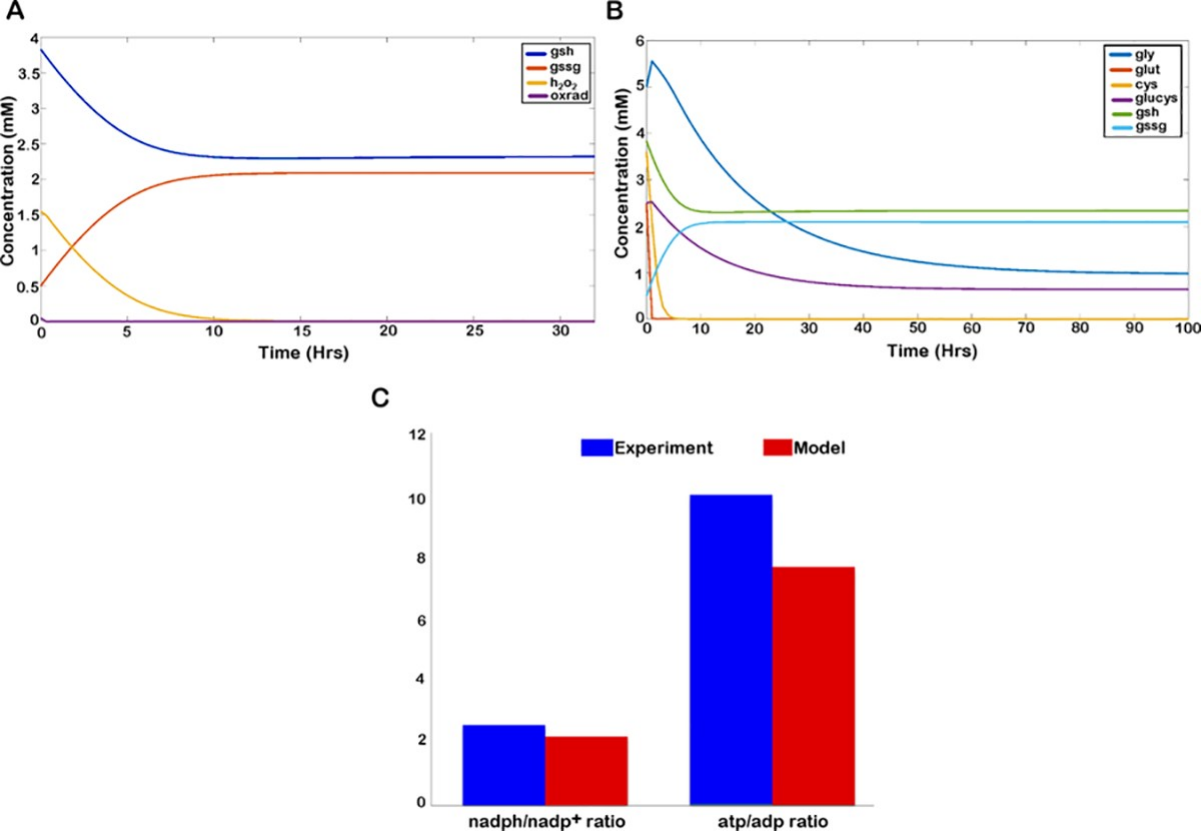
Model properties. A. The temporal plot of *gsh*, *gssg*, *h*_*2*_*o*_*2*_, and *oxrad*. B. Temporal plot of the tri-peptides cystine (*cys*), glutamate (*glut*) and glycine (*gly*) and glutamyl-cysteine (*glucys*) along with changes in *gsh* and *gssg*. C. Comparison of experimentally obtained range of *nadph/nadp*^*+*^ and *atp/adp* ratio with values obtained through model simulation.

In order to validate the model properties, we used available experimental data for comparison with model outcomes. Under normal physiological conditions, cells maintain a reduced redox state with a high *nadph/nadp*^*+*^ ratio. *Nadp*^*+*^ primarily acts as an electron donor in metabolic pathways, and a low *nadph/nadp*^*+*^ depicts an enhanced oxidative state of the cell. A properly functional ROS machinery of a normal cell maintains a balanced *nadph-nadp*^*+*^ pool, which is kept in a significantly reduced state by maintaining a high *nadph/nadp*^*+*^ ratio [36]. As such, we validated the model with experimentally determined *nadph/nadp*^*+*^ ratio [37], which ranged between 2.5–3 (Fig 3C). The average *atp/adp* ratio under functional glycolysis was also measured (Fig 3C). Non-proliferative cells maintain a very high cytosolic *atp/adp* ratio by metabolizing respiratory substrates, which essentially inhibits glycolysis [38]. However, with the initiation of cellular activity, glycolysis is initiated, which maintains an average cytosolic *atp/adp* ratio between 2.6–10 [39, 40] that has been captured through our simulation as well.

Furthermore, the model was simulated with an initial *gsh* concentration of 3.83mM, as has been reported in the case of glial cells [34]. Our simulations showed that the model was capable of maintaining *h*_*2*_*o*_*2*_ concentration within a biologically feasible range of ~4μM while maintaining a value of ~2.33mM of *gsh* at steady-state (Fig 3A), which is in agreement with the reported basal cytoplasmic concentration of *gsh* [41]. The *gsh* metabolizing cells maintain a negligibly small cytosolic pool of *cystine* which is readily reduced to *cysteine*, the rate limiting amino-acid of *gsh* biosynthesis [42]. This property has been captured in the model, where the *cystine* concentration is readily reduced to micromolar levels upon initiation of *gsh* metabolism (Fig 3B). Also, *cystine* import is coupled with *glutamate* through cystine-glutamate antiporter which allows the release of *glutamate* to extracellular matrix upon cystine uptake. Due to its role as a neurotransmitter, the extracellular concentration of *glutamate* in brain is tightly controlled and maintained at a micromolar concentration ranging between 2–9μM [43]. We could observe the diminished level of extracellular glutamate through our simulation (Fig 2B) based on temporal data available for astrocytes, which has been further used for parameter estimation of glutamate exchange reaction. Note that the following validations have been made under normal glial condition. High *nadph/nadp*^*+*^ and *atp/adp* ratios are characteristic of any normal proliferating cell, although the values for the ratios are specific to simulation conditions. The external glutamate concentration holds true for extracellular matrix of brain cells. The concentration of *gsh* is specific to glial cells and the concentration of all other metabolites upon simulation are also characteristic of glial cells.

### 2.6. Sensitivity analysis

Extended Fourier Amplified Sensitivity Test (eFAST) algorithm was used for identifying sensitive parameters to the system. The algorithm makes use of the variance decomposition method to predict the sensitivity of parameters in a nonlinear non-monotonic system. First-order sensitivity index S_i_, and total order sensitivity index, S_Ti_, were calculated for different transient time points and steady states of *gsh*, *gssg*, *h*_*2*_*o*_*2*_ and *oxrad*. Sensitivity plots of parameters with a p-value < 0.05 for *gsh* (reduced glutathione), *gssg* (oxidized glutathione), *h*_*2*_*o*_*2*_ (hydrogen peroxide) and *oxrad* (oxygen radical) are provided in S3 Fig of S1 Text. The analysis was repeated for high intracellular oxygen demand and multiple mutation conditions to check the sensitivity of parameters at different conditions.

### 2.7. Parameter variation

Parameter variation analyses for a single parameter and two parameters were performed to understand their effect on the respective variables. Parameters were varied over a feasible biological range at any given time point. For most of the instances, changes in the enzyme concentrations have been introduced by varying the V_max_ of the enzyme, as V_max_ is determined by the enzyme concentration and substrate availability. For two-parameter variation analysis, two parameters were simultaneously varied and the results were plotted as 3D surface plots to show their effect on the respective variable.

### 2.8. Changing oxygen demand: The creation of hypoxia situation

The oxygen uptake by cells is approximated by Michaelis-Menten kinetics in the model where V_max_ represents the rate of oxygen uptake by the cell and K_m_ represents the affinity of the cell for extracellular oxygen. Low K_m_ signifies a high affinity for oxygen. The value of K_m_ for extracellular oxygen had been varied to create low to high intracellular oxygen demand. A low K_m_ value created high oxygen demand within the cell which eventually created a hypoxic condition in the extracellular environment. Hence, a lower K_m_ value also represented a hypoxic condition.

To create the hypoxic scenario, oxygen uptake rates of the cell was enhanced by reducing the K_m_ of Oxygen ($k_{m}^{O_{2}}$). The initial K_m_ value for oxygen uptake was 164*mM* for the normal condition. This was reduced down a very low value of 1*mM*, which signifies a high affinity for the substrate, which in the present model is the extracellular oxygen. The hypoxic condition which resulted was a consequence of the rapid uptake of oxygen by the cell. Hence, hypoxia was induced as a consequence of rapid metabolism of the cells.

### 2.9. Creation of glioma-like situation

In order to create a glioma like scenario in the model, we introduced changes in the values of multiple parameters to induce the change in activities of the respective enzymes. The selection of these parameters was made based on literature evidences of their malfunctioning in gliomas and also by the analysis of sensitive parameters. Differential regulations of the enzymes NADPH oxidase (*NOX*) [44, 45], glutathione peroxidase (*GTHP*) [33, 46] and glutathione oxidoreductase (*GTHO*) [47] have been reported in the literature. The sensitivity analysis further added to the understanding of the parameters which govern these changes. These include $V_{m}^{NOX}$, $V_{m}^{GTHO}$, $V_{m}^{GTHP}$ and $k_{m}^{O_{2}}$ where $k_{m}^{O_{2}}$ has been considered to alter the oxygen demand of the cell as described in the earlier section. An increase in *h*_*2*_*o*_*2*_ concentration was considered as a signature to ensure that the model represents a glioma-like situation [48, 49].

## 3. Results

### 3.1. Dynamics of cells under normoxic conditions

We illustrate the temporal behavior of glutathione (reduced and oxidized), hydrogen peroxide (*h*_*2*_*o*_*2*_) and oxygen radicals (*oxrad*) over time in the normal scenario. The chemical kinetics of the enzyme *GTHP* has been included which uses *h*_*2*_*o*_*2*_ and *gsh* as the substrate to yield*gssg*. From simulations we observe that the model is capable of maintaining *h*_*2*_*o*_*2*_ concentration within a biologically feasible range of ~4 **μ***M* which was initiated at a high *h*_*2*_*o*_*2*_ concentration of 1.5mM (Fig 3A), while there is a decrease in the *gsh* concentration (from 3.83*mM* to 2.33*mM*) and increase in the *gssg* concentration (from 0.5*mM* to 2.09*mM*) at steady state. Meanwhile, the oxygen radicals (*oxrad*) generated as an action of NADPH oxidase (*NOX*) is readily metabolized into *h*_*2*_*o*_*2*_ due to high activity of superoxide dismutase (*SOD*) as has been reported and considered in the model ($V_{m}^{SOD}$ = 11.4*10^3^ *mM hr*^*-1*^). Hence, a consistent reduced level of *oxrad* is observed through our simulations.

The *nadph/nadp*^*+*^ and *atp/adp* ratios of the model have also been considered. As has been observed experimentally, a proliferating cell maintains a high *nadph/nadp*^*+*^ ratio to maintain its redox balances and high *atp/adp* ratio to suffice its proliferative requirements. In Fig 3C, we have compared the experimentally reported values of *nadph/nadp*^*+*^ [37] and average *atp/adp* [39, 40] ratios with the simulated values which are comparable. The dynamics of the components of tripeptide that result in the formation of *gsh* is dictated by the *gsh-gssg* cycle. Fig 3B shows the changes in the intracellular concentration of cystine (*cys*), glutamate (*glut*), glycine (*gly*) and glutamyl-cysteine (*glucys*) corresponding to changes in *gsh* and *gssg* over time. Simulation results show that in response to high oxidant (*h*_*2*_*o*_*2*_) concentration within the cell, available intracellular cystine and glutamate is used for the production of glutamyl-cysteine, which subsequently forms a complex with glycine to produce *gsh*. *gsh* then enters the *gsh-gssg* cycle where *gsh* and *h*_*2*_*o*_*2*_ are used as a substrate to produce *gssg*. As such, we observe a decline in the concentration of all other metabolites except for *gssg*, which is produced in response to nullify the high *h*_*2*_*o*_*2*_ concentration. The intracellular concentration of cystine and glutamate remain limiting in a normal scenario.

#### 3.1.1. Parameter variation of sensitive parameters

Sensitivity analysis yielded a set of parameters crucial for determining the *h*_*2*_*o*_*2*_ level and the regulation of the *gsh-gssg* cycle. Enzymes *GTHP*, *NOX* and *GTHO* are observed to be most sensitive in determining model properties. Changes in the uptake rate of oxygen also affected the model dynamics. Varying parameters for these enzymes show interesting results, which are discussed in the subsequent subsections.

$V_{\max}\;\mathrm{of}\;\mathrm{Glutathione}\;\mathrm{Peroxidase}\;\left(V_{m}^{\mathrm{GTHP}}\right):$

**Fig 4:**
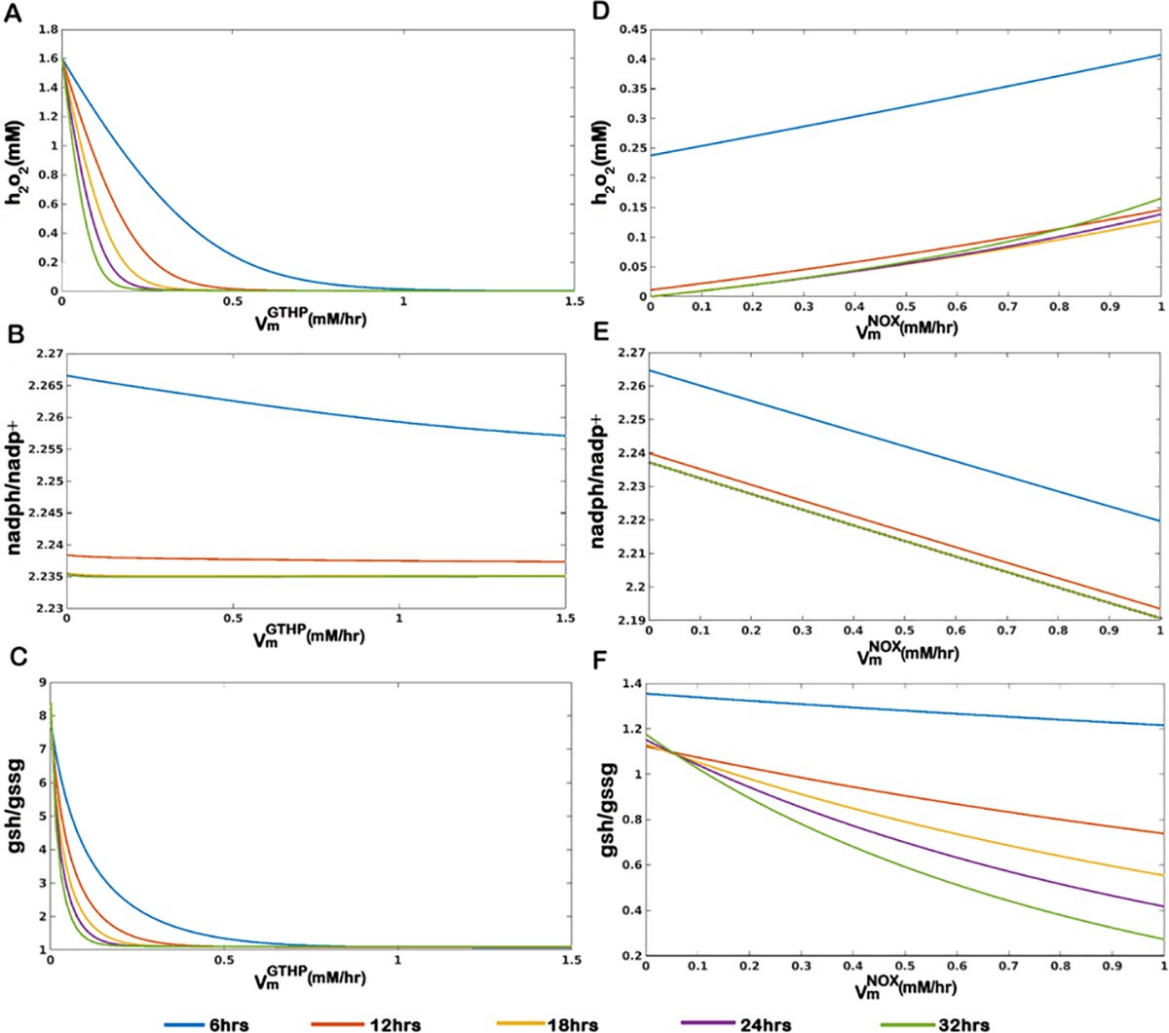
Parameter variation plot for (A-C) V_max_ of GTHP ($V_{m}^{GTHP}$) and (D-F) V_max_ of NOX *($V_{m}^{NOX}$)* under the normal condition at different time points.

The enzyme *GTHP* is crucial in neutralizing *h*_*2*_*o*_*2*_ with the help of *gsh* which itself converts to *gssg* converting *h*_*2*_*o*_*2*_ into *h*_*2*_*o*. The V_max_ of the reaction is an important determinant of the rate of conversion of *h*_*2*_*o*_*2*_. From our analysis, we could see that an increase in the $V_{m}^{GTHP}$ (from 0.001 *mM hr*^*-1*^ to 1.5 *mM hr*^*-*1^) resulted in a reduced level of *h*_*2*_*o*_*2*_. Simulations were performed at different time points (6hrs, 12hrs, 18hrs, 24hrs, and 32 hrs) and a similar trend is observed for all time points. A sharp decrease is observed at a V_max_ value between 0.1 *mM hr*^*-1*^ to 0.3 *mM hr*^*-1*^ when the *h*_*2*_*o*_*2*_ concentration is maintained at micromolar concentration, which initiated at a millimolar concentration (Fig 4A). However, the corresponding *nadph/nadp*^*+*^ ratio remains unaltered which suggests that the redox balance of the system largely remains unaffected by the change in $V_{m}^{GTHP}$ although the *gsh/gssg* ratio reduces significantly (Fig 4B and 4C). It is important to mention that the normal physiological expression of *GTHP* ranges between 0.2874 to 2.697 mM/hr (S1 Table of S1 Text) and interesting dynamics in *h*_*2*_*o*_*2*_ level, *nadph/nadp*^*+*^ and *gsh/gssg* ratios are only observable for a very low value of $V_{m}^{GTHP}$. By varying the value of $V_{m}^{GTHP}$ from 0.001 *mM hr*^*-1*^ to 1.5 *mM hr*^*-1*^ we observe that high *GTHP* activity maintains a steady and low micromolar concentration of *h*_*2*_*o*_*2*_, but *h*_*2*_*o*_*2*_ starts accumulating as the activity lowers, which might induce oxidative damage to the cell. This observation can be compared with the diminished level of *GTHP* in brain tumors [50] and can be interpreted as a characteristic for gliomas with a very low expression of *GTHP*.

$V_{max}\;of\;NADPH\;Oxidase\;(V_{m}^{NOX}):$

NADPH Oxidase catalyzes the production of oxygen free radicals from available oxygen using NADPH reduction. We could observe that an increase in the $V_{m}^{NOX}$ (from 0.0001 *mM hr*^*-1*^ to 1 *mM hr*^*-1*^) results in an increase in the *h*_*2*_*o*_*2*_ concentration for the specified time points (6hrs, 12hrs, 18hrs, 24hrs and 32 hrs). A marked decrease in the *nadph/nadp*^*+*^ ratio and *gsh/gssg* ratio is observed at later time points (24hrs and 32hrs) with the increase in V_max_ value (Fig 4D, 4E and 4F). This could be interpreted as one of the important factors determining the cancerous transformation of the glial cells.

$Two\;parameter\;variation\;of\;V_{m}^{GTHP}\;and\;V_{m}^{GTHO}:$

**Fig 5:**
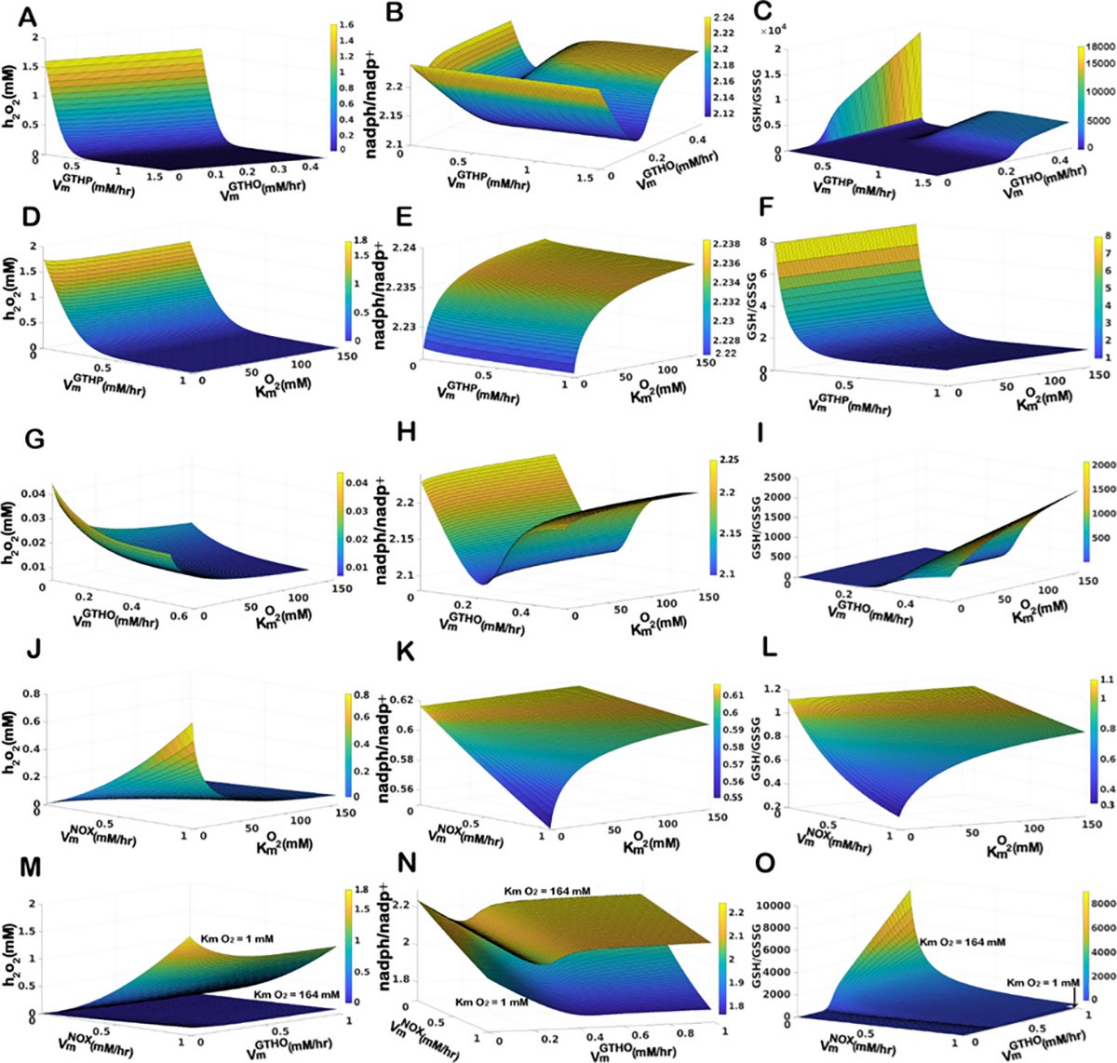
Surface plots of two parameter variation for the sensitive parameters on *h*_*2*_*o*_*2*_ level, *nadph/nadp*^*+*^ ratio, and *gsh/gssg* ratio. (A-C) Effect of variations in $V_{m}^{GTHP}$ and $V_{m}^{GTHO}$ under normoxic conditions ($k_{m}^{O_{2}}$ = 164*mM*). (D-F) Effect of variation in $V_{m}^{GTHP}$ under changing oxygen demand (164*mM* ≥ $k_{m}^{O_{2}}$≥ 1*mM*). (G-I) Effect of variation in $V_{m}^{GTHO}$ under changing oxygen demand (164*mM* ≥ $k_{m}^{O_{2}}$≥ 1*mM*). (J-L) Effect of variation in $V_{m}^{NOX}$ under changing oxygen demand (164*mM* ≥ $k_{m}^{O_{2}}$ ≥ 1*mM*). (M-O) Combined effect of simultaneous variation in $V_{m}^{NOX}$ and $V_{m}^{GTHO}$ under $k_{m}^{O_{2}}$ = 164*mM* and $k_{m}^{O_{2}}$ = 1*mM*.

We illustrate in (Fig 5A–5C), the effect of simultaneous change in $V_{m}^{GTHP}$ and $V_{m}^{GTHO}$ on *h*_*2*_*o*_*2*_ level, *nadph/nadp*^*+*^ and *gsh/gssg* ratios. The *h*_*2*_*o*_*2*_ level is reduces with increasing activity of $V_{m}^{GTHP}$, although change in the kinetics of *GTHO* does not affect (Fig 5A). When *nadph/nadp*^*+*^ is taken into account, we observe that at a very low $V_{m}^{GTHO}$, the effect of $V_{m}^{GTHP}$ remains minimum. With a gradual increase in the $V_{m}^{GTHO}$ there is a reduction in the *nadph/nadp*^*+*^ ratio, which furrows deeper at a higher value of $V_{m}^{GTHP}$. The enzyme *GTHO* facilitates the reduction of *gssg* into *gsh* in a *nadph-nadp*^*+*^ dependent manner and hence with an increasing enzyme availability and activity the *nadph/nadp*^*+*^ ratio reduces. Here we have considered the dynamics of *nadph/nadp*^*+*^ at 12hrs and could observe that the initial dynamics are dependent on $V_{m}^{GTHO}$. At high $V_{m}^{GTHP}$, with increasing value of $V_{m}^{GTHO}$ there is a decrease in the ratio (Fig 5B) which is due to the active involvement of the enzyme in nullifying the persistent levels of the substrate, *h*_*2*_*o*_*2*_. We observe the decrease in the ratio till $V_{m}^{GTHO}$ reaches a value of 0.2 *mM hr*^*-1*^ at 12 hours. However, the decrease is compensated back with further increase in the V_max_. As a high level of *h*_*2*_*o*_*2*_ is metabolized to a non-toxic micromolar level, we observe from our model simulation that a V_max_ of 0.2 *mM hr*^*-1*^ for the enzyme GTHO is sufficient to metabolize the persisting levels of *h*_2_*o*_2_ at 12 hours. At a V_max_ value lower than this the enzyme becomes the limiting factor and beyond this value, the activity of the enzyme is limited by the availability of the substrate (*h*_*2*_*o*_*2*_). Meanwhile, at lower values of $V_{m}^{GTHP}$(<0.5 *mM hr*^*-1*^), persisting level of *h*_*2*_*o*_*2*_ at 12hrs is higher due to the slow activity of *GTHP* (Fig 5B). The substrate remains available for *GTHO* activity which continues to metabolize the conversion (at higher $V_{m}^{GTHO}$ which was limited by substrate concentration in the earlier case) and *nadph* pool diminishes resulting into a reduction in *nadph/nadp*^*+*^ ratio. The dip in the *nadph/nadp*^*+*^ ratio is compensated by the action of other reactions that replenish *nadph* pools represented in the model as *l*_*nadph*_. Trends are similar for *gsh*/*gssg* ratio which is dependent on the substrate availability (*h*_*2*_*o*_*2*_) except at a very low value of $V_{m}^{GTHP}$(0.001 *mM hr*^*-1*^) which limits the conversion of *gsh* to *gssg* thereby resulting in an accumulation of *gsh* and leading to very high *gsh/gssg* ratios. *h*_*2*_*o*_*2*_ level persists in the sub-micromolar range (~0.045 *mM*) at a V_max_ of 0.5 *mM hr*^*-1*^ for *GTHP*, which is subsequently metabolized to micromolar levels (0.002 *mM*) with further increase in V_max_ (Fig 5A). This governs the change in *gsh/gssg* ratio and an increase in both $V_{m}^{GTHP}$ and $V_{m}^{GTHO}$ coordinates to metabolize *h*_*2*_*o*_*2*_ till it reaches micromolar levels by cyclic production and consumption of *gsh* and *gssg*. As *h*_*2*_*o*_*2*_ reaches a micromolar level the activity of *GTHP* is limited due to *h*_*2*_*o*_*2*_ availability whereas *gsh* production continues by the action of *GTHO* till *gssg* reaches a basal level. At this point we observe an increase in the *gsh/gssg* ratio (Fig 5C).

### 3.2. Dynamics of cells under high oxygen demand: induction of hypoxia in the microenvironment

To mimic the high oxygen demand during cancerous transformation we tuned the model parameter $k_{m}^{O_{2}}$ which determines the affinity for the substrate. A decrease in the K_m_ value implies an increase in the affinity for the substrate. To represent an increased affinity of the cell for oxygen, lowering values of K_m_ is tried ($k_{m}^{O_{2}}$ = 164, 50, 10 and 1*mM*) and as the value lowers, the concentration of external oxygen reduces creating a mild to severe hypoxic condition (S4 Fig of S1 Text). Parameter variation of sensitive parameters is tried for different affinities for oxygen and results are discussed below.

#### 3.2.1. Effect of Changing Glutathione Peroxidase (GTHP) enzymatic activity

With the change in dynamics of *h*_*2*_*o*_*2*_, *nadph/nadp*^*+*^ and *gsh/gssg* ratios with changing $V_{m}^{GTHP}$ and $k_{m}^{O_{2}}$, we observe that *h*_*2*_*o*_*2*_ concentration (Fig 5D) and *gsh/gssg* ratio (Fig 5F) primarily depends on $V_{m}^{GTHP}$ and remain unaffected by the change in $k_{m}^{O_{2}}$. The enzyme *GTHP* metabolizes the conversion of *h*_*2*_*o*_*2*_ into water by oxidizing *gsh* to *gssg* and hence the enzyme concentration directly affects the *h*_*2*_*o*_*2*_ concentration and *gsh/gssg* ratio. *nadph/nadp*^*+*^ ratio however, depend on $k_{m}^{O_{2}}$ and remain unaltered with change in the $V_{m}^{GTHP}$(Fig 5E). We infer that high levels of oxidants created in response to increased uptake of oxygen (which eventually creates a hypoxic microenvironment for the cell) can be dealt with increased activity of *GTHP* within the cell, but at a cost of reduced *nadph/nadp*^*+*^ and *gsh/gssg* ratios.

#### 3.2.2 Effect of Changing Glutathione Oxidoreductase (GTHO) enzymatic activity

At 12 hours, *h*_*2*_*o*_*2*_ is maintained between sub-micromolar to micromolar levels (0.045*mM* to 0.005*mM*) for varying levels of $V_{m}^{GTHO}$ and $k_{m}^{O_{2}}$. The concentration of *h*_*2*_*o*_*2*_ reduces with increasing $V_{m}^{GTHO}$. Simultaneously with increasing $k_{m}^{O_{2}}$, a further reduction in *h*_*2*_*o*_*2*_ level is observed (Fig 5G). The *nadph/nadp*^*+*^ ratio shows a dip at V_max_ value of 0.2 *mM hr*^*-1*^ for GTHO which is compensated back with further increase in the V_max_ (Fig 5H). We plotted the temporal plots of varying $V_{m}^{GTHO}$ at high oxygen demand ($k_{m}^{O_{2}}$ = 1*mM*) and checked the *nadph/nadp*^*+*^ ratios at different time points (S5 Fig of S1 Text). We infer from our model simulations that the *nadph/nadp*^*+*^ ratio in the model depends upon two factors: (i) concentration of the enzyme *GTHO* available to convert the *gssg* into *gsh* in order to maintain the *gsh*-*gssg* cycle necessary for neutralizing *h*_*2*_*o*_*2*_ levels (ii) the amount of oxidant (*h*_*2*_*o*_*2*_) concentration persisting at any point of time. The *gsh*/*gssg* ratio shows different patterns at different values of the two parameters (Fig 5I). At low values of $V_{m}^{GTHO}$, the enzymatic activity is limited and hence the conversion of *gssg* to *gsh* is slower. With a low $V_{m}^{GTHO}$ (< 0.2*mM*), *gssg* accumulates and the difference in *gsh* and *gssg* concentration reduces resulting in low *gsh/gssg* ratio. As the V_max_ increases, the conversion of *gssg* to *gsh* enhances which adds to the *gsh* production pool and the difference in *gsh* and *gssg* concentration widens giving high values of *gsh/gssg* ratio. Adding to it is the effect of oxygen within the cell. As $k_{m}^{O_{2}}$ increases, the oxidant production in limited (due to limited substrate availability for *NOX* and *SOD*) and hence the conversion of *gsh* to *gssg* is reduced leading to further increase in the *gsh/gssg* ratio.

#### 3.2.3 Effect of changing NADPH Oxidase (NOX) enzymatic activity

Characteristic changes in *h*_*2*_*o*_*2*_, *nadph/nadp*^*+*^ ratio and *gsh/gssg* ratio with changing $V_{m}^{NOX}$ is observed under normoxic conditions. When we simulate the variation of the parameter over varying $k_{m}^{O_{2}}$, the concentration of *h*_*2*_*o*_*2*_ takes a leap as $V_{m}^{NOX}$ increases and $k_{m}^{O_{2}}$ decreases (Fig 5J). At smaller K_m_ value, cellular uptake of oxygen increases and an increase in $V_{m}^{NOX}$ ensures rapid metabolism of oxygen into free radicals. These free radicals are readily being metabolized into *h*_*2*_*o*_*2*_ due to the high $V_{m}^{SOD}$ which catalyzes the conversion. Hence, with increasing activity of *NOX* the free radicals so formed are directed towards the production of *h*_*2*_*o*_*2*_. An inverse pattern of *nadph/nadp*^*+*^ and *gsh/gssg* ratio is observed in response to the changing *h*_*2*_*o*_*2*_ concentration (Fig 5J) due to the activity of the *gsh-gssg* cycle (Fig 5K and 5L).

We infer from the above simulations that *GTHP* is an important determinant of *h*_*2*_*o*_*2*_ levels in the cell. An increase in the activity of *GTHP* is helpful in reducing the *h*_*2*_*o*_*2*_ levels to a feasible range without affecting the *nadph/nadp*^*+*^ ratio much although it alters the *gsh/gssg* ratio. The effect of changing affinity for oxygen is nullified with a change in $V_{m}^{GTHP}$. We attempted varying the activity of *GTHP* with other sensitive parameters only to observe a reduction in the *h*_*2*_*o*_*2*_ while maintaining the redox balance of the cell within a feasible range. As such we propose here that an increase in the activity of *GTHP* is desirable for anti-oxidant therapy as it reduces the *h*_*2*_*o*_*2*_ levels irrespective of oxygen demand simultaneously lowering the *gsh/gssg* ratio which is considered an initiation factor in the induction of apoptosis.

While *GTHP* can be used for anti-oxidant therapy, analyses with *NOX* and *GTHO* suggest that they are favorable targets for pro-oxidant therapies. An increase in *NOX* activity at high oxygen demand clearly increases *h*_*2*_*o*_*2*_ production along with simultaneous lowering of *nadph/nadp*^*+*^ and *gsh/gssg* ratio indicating cellular toxicity and initiation of apoptotic pathways. Changes in *GTHO* activity have differing patterns. Given the initial concentration of *h*_*2*_*o*_*2*_ in the model, a decrease in *h*_*2*_*o*_*2*_ concentration is observed with increasing *GTHO* activity. However, *nadph/nadp*^*+*^ and *gsh/gssg* ratio at around a V_max_ value of 0.2 *mM hr*^*-1*^ of *GTHO* shows an ideal condition to initiate cellular toxicity. We further analyzed the effect of the two enzymes in reducing redox potential and *gsh/gssg* ratio of the cell at different oxygen demand of the cell.

An increase in *NOX* and *GTHO* activity has a synergistic effect in increasing the *h*_*2*_*o*_*2*_ concentration (Fig 5M) and lowering the *nadph/nadp*^*+*^ ratio (Fig 5N) under high oxygen demand. *gsh/gssg* ratio, however, lowers down with increasing activity of *NOX*, which otherwise increases with increasing *GTHO* activity at low *NOX* activity (<0.3 *mM hr*^*-1*^) (Fig 5O).

### 3.3. Cellular behavior with multiple mutations

From the previous set of analyses, we observe that the system shows a characteristic change in its behavior for changes in sensitive parameters which are related to the oxidant and anti-oxidant production. The system remained robust for most of the other parameter changes. The sensitivity of the *h*_*2*_*o*_*2*_ increased for *GTHO* during high oxygen demand which otherwise remains unaffected in normoxia conditions. Also, the sensitivity of *h*_*2*_*o*_*2*_ increased for *NOX* during high oxygen demand. Parameter values to $V_{m}^{NOX}$, $V_{m}^{GTHO}$, $V_{m}^{GTHP}$ and $k_{m}^{O_{2}}$ were changed to 1*mM hr*^*-1*^, 0.2 *mM hr*^*-1*^, 0.19 *mM hr*^*-1*^and 1 *mM* from 0.0468 *mM hr*^*-1*^, 0.5 *mM hr*^*-1*^, 0.00216 *mM hr*^*-1*^and 164*mM* respectively to create the glioma scenario. Temporal plots generated for this model show an excess increase in *h*_*2*_*o*_*2*_ concentration due to limited regulation by *gsh* and *gssg*. A decline in *nadph/nadp*^*+*^ and *gsh/gssg* ratio is also observed.

**Fig 6:**
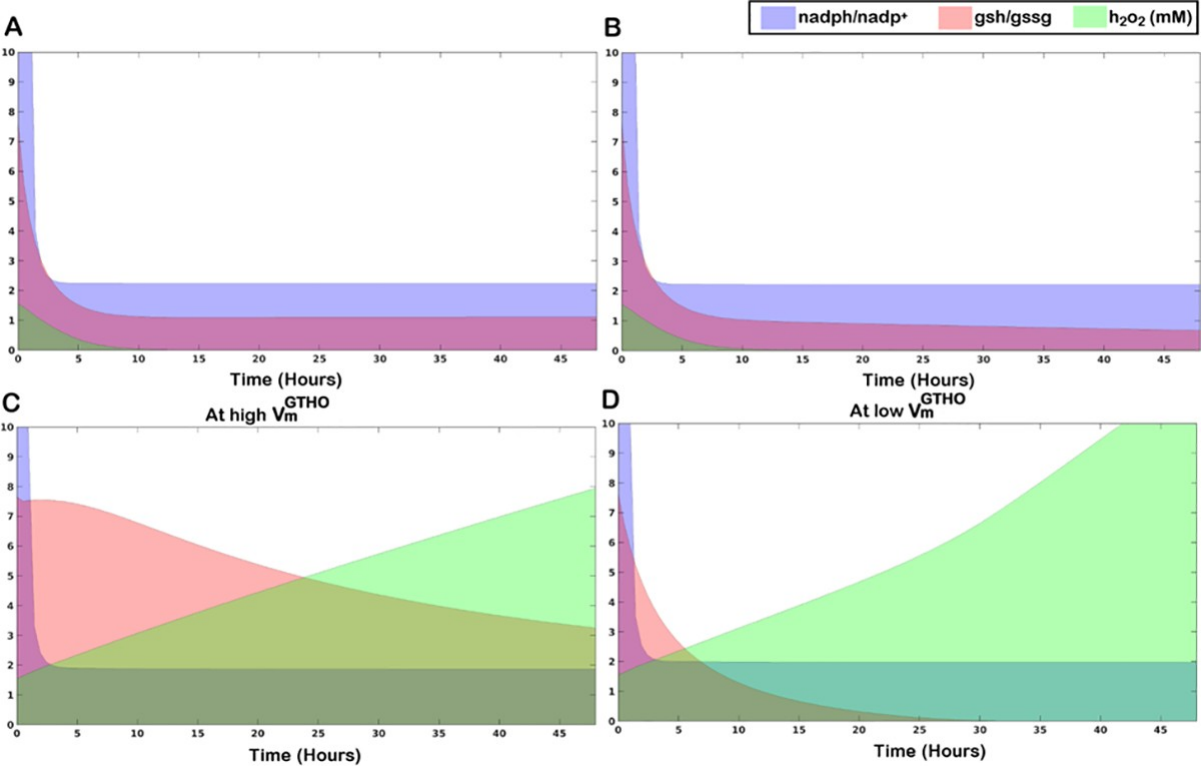
Temporal area plots of changing *nadph/nadp*^*+*^ and *gsh/gssg* ratios along with change in *h*_*2*_*o*_*2*_ concentration. A. Normal condition, B. Hypoxia, C. Glioma at high $V_{m}^{GTHO}$(0.19 *mM hr*^*-1*^), D. Glioma at low $V_{m}^{GTHO}$ (0.001 *mM hr*^*-1*^).

With an observable difference in the cellular redox status and the ROS level, we try to understand if the changes in *nadph/nadp*^*+*^ and *gsh/gssg* ratios associated with the changes in *h*_*2*_*o*_*2*_ level in the glioma scenario can be used to determine the pro-apoptotic or anti-apoptotic fates of the cell. Temporal dynamics of the changes in *h*_*2*_*o*_*2*_ level and the two ratios for Normal, Hypoxia and Glioma conditions for a duration of 48hrs are shown in Fig 6. Under normal conditions, *h*_*2*_*o*_*2*_ levels are readily reduced to micromolar concentration (4**μ**M) and the resulting values of *nadph/nadp*^*+*^ and *gsh/gssg* ratios at steady state are 2.63 and 1.2 (Fig 6A). The values mostly remain unaltered during a shift to hypoxia except for a slight reduction in the *gsh/gssg* ratio over time (Fig 6B). Changes are however distinct in case of glioma. Reports suggest that different GBM cell lines have different level of *GTHO* expression and overexpression of *GTHO* mediates drug resistance in these cells. Whereas, *GTHO* knockdown resensitize *GTHO* overexpressed cells to drug treatment [47]. We corroborated this understanding to our model simulations to observe the effect of varying *GTHO* on the thiol and redox ratios and *h*_*2*_*o*_*2*_ concentration. We created both the conditions: high and low expression of *GTHO* in the model. We observe a characteristic difference in the *gsh/gssg* ratio and *h*_*2*_*o*_*2*_ level in the two scenarios. Under high *GTHO* expression ($V_{m}^{GTHO}$ = 0.19 *mM hr*^*-1*^), the *gsh/gssg* ratio remains higher than the normal (Fig 6C) whereas, under low *GTHO* expression ($V_{m}^{GTHO}$ = 0.001 *mM hr*^*-1*^) the ratio diminishes to a very low value of 0.01 with an abrupt increase in *h*_*2*_*o*_*2*_ level (Fig 6D). Through the simulations, we propose that at high values of $V_{m}^{GTHO}$, the increased level of *gsh/gssg* ratio helps the cell to evade programmed cell death which would otherwise lead to apoptosis by the induction of toxicity due to uncontrolled increase in *h*_*2*_*o*_*2*_ levels. However, at lower values of $V_{m}^{GTHO}$, a sharp decline in the *gsh/gssg* ratio drives the cell towards an apoptotic fate. As such employing an anti-oxidant approach at high $V_{m}^{GTHO}$ and a pro-oxidant approach at low $V_{m}^{GTHO}$ will provide a better surveillance strategy to eliminate cancer cell progression. We observe only a slight difference in the *nadph/nadp*^*+*^ ratio under present simulation conditions. Regulation of *nadph/nadp*^*+*^ ratio, however, can be employed to facilitate the pro- or anti-oxidant approach by modulating *nadph/nadp*^*+*^ ratio either by inhibition or activation of NAD kinase, a potent regulator of the *nadph*-*nadp*^+^ pool within the cell [51, 52].

**Fig 7:**
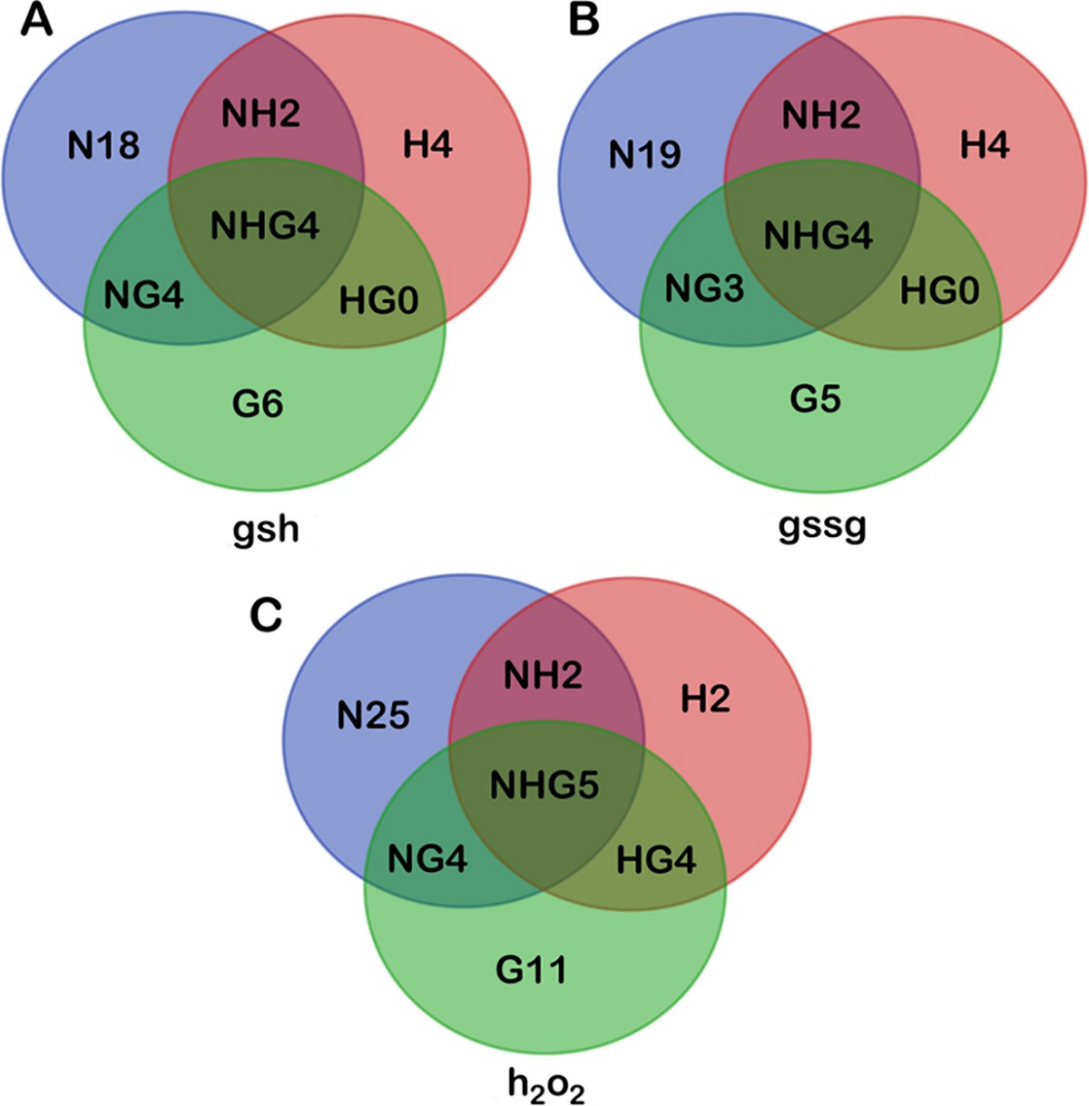
Comparison of sensitive parameters in Normal, Hypoxia and Gliomas for the variables *gsh*, *gssg* and *h*_*2*_*o*_*2*_. The abbreviations used here are **N:** Normal, **H:** Hypoxia, **G:** Gliomas, **NH:** Normal and Hypoxia, **NG:** Normal and Gliomas, **HG:** Hypoxia and Gliomas and **NHG:** Normal, Hypoxia and Gliomas. The sensitive parameters for each variable under each condition have been tabulated in Table 1.

To identify parameters, which influence the cellular properties under these different conditions, sensitivity analyses are performed for the model with high oxygen demand (hypoxia scenario, by changing $k_{m}^{O_{2}}$) and for the model with multiple mutations (glioma scenario). A comparison of the sensitive parameters (p-value <0.05) for the variables *h*_*2*_*o*_*2*_, *gsh* and *gssg* for the three conditions: normal, hypoxic and glioma show common and unique sensitive parameters for each scenario (Fig 7). The kinetics of the enzyme *GTHP* remains crucial in all three scenarios owing to its direct involvement in the production of *gsh*. Apart from the parameters which directly regulate the variables, a few indistinct parameters are found to be sensitive for the different scenarios. Analyses of these parameters show that the influence exerted by them on the variables is at micromolar and nanomolar concentrations for the present simulation conditions. The set of parameters which are unique and common to the three different simulation scenarios are shown in Fig 7 and the detail descriptions are provided in Table 1. We suggest that regulation of these parameters might help in facilitating pro- or anti-oxidant therapeutic strategies.

**Table 1:** List of sensitive parameters for *gsh*, *gssg* and *h*_*2*_*o*_*2*_ under normal, hypoxic and glioma scenarios.

***gsh***	
**N18**	$V_{m}^{PGI}$, $k_{3php}^{PST}$, $k_{atp}^{GS}$, $k_{m}^{O_{2}}$, $k_{i(3pg)}^{PGCDH}$, $k_{atp}^{GCL}$, $k_{i(cys)}^{CR}$, $V_{m}^{GHMT}$, $k_{cys}^{CR}$, $k_{f6p}^{PFK}$, *d*_*oxy*_, $k_{i(glut)}^{PST}$, $V_{m}^{GLUTEX}$, $V_{m}^{PSP}$, $k_{i(gap)}^{GAPDH}$, $k_{gap}^{GAPDH}$, $k_{i(gly)}^{GS}$, $V_{m}^{PFK}$
**H4**	$k_{i(atp)}^{HK}$, $k_{oxrad}^{SOD}$, $k_{i(atp)}^{PFK}$, $k_{O_{2}}^{NOX}$
**G6**	$k_{i(gssg)}^{GTHO}$, $V_{m}^{NOX}$, $V_{m}^{GS}$, *l*_*nadp*_, *l*_*nadph*_, *d*_*nadph*_
**NH2**	$k_{adp}^{PGK}$, $V_{m}^{GCL}$
**HG0**	-
**NG4**	$V_{m}^{GTHO}$, $k_{gssg}^{GTHO}$, $k_{i(atp)}^{GS}$, $k_{nadph}^{GTHO}$
**NHG4**	$k_{i(h_{2}o_{2})}^{GTHP}$, $V_{m}^{GTHP}$, $k_{h_{2}o_{2}}^{GTHP}$, $k_{gsh}^{GTHP}$
***Gssg***	
**N19**	$V_{m}^{PGI}$, $k_{3php}^{PST}$, $k_{atp}^{GS}$, $k_{m}^{O_{2}}$, $k_{i(3pg)}^{PGCDH}$, $k_{atp}^{GCL}$, $k_{i(cys)}^{CR}$, $V_{m}^{HK}$, $V_{m}^{GHMT}$, $k_{cys}^{CR}$, $k_{f6p}^{PFK}$, *d*_*oxy*_, $k_{i(glut)}^{PST}$, $V_{m}^{GLUTEX}$, $V_{m}^{PSP}$, $k_{i(gap)}^{GAPDH}$, $k_{gap}^{GAPDH}$, $k_{i(atp)}^{GS}$, $V_{m}^{PFK}$
**H4**	$k_{i(atp)}^{HK}$, $k_{oxrad}^{SOD}$, $k_{i(atp)}^{PFK}$, $k_{O_{2}}^{NOX}$
**G5**	$k_{i(gssg)}^{GTHO}$, $V_{m}^{NOX}$, *l*_*nadp*_, *l*_*nadph*_, *d*_*nadph*_
**NH2**	$k_{adp}^{PGK}$, $V_{m}^{GCL}$
**HG0**	-
**NG3**	$V_{m}^{GTHO}$, $k_{gssg}^{GTHO}$, $k_{nadph}^{GTHO}$
**NHG4**	$k_{i(h_{2}o_{2})}^{GTHP}$, $V_{m}^{GTHP}$, $k_{h_{2}o_{2}}^{GTHP}$, $k_{gsh}^{GTHP}$
***h***_**2**_***o***_**2**_	
**N25**	$V_{m}^{PGI}$, $k_{3php}^{PST}$, $k_{atp}^{GS}$, $k_{m}^{O_{2}}$, $k_{i(3pg)}^{PGCDH}$, *d*_*nadh*_, $k_{atp}^{GCL}$, $k_{i(cys)}^{CR}$, $V_{m}^{HK}$, *d*_*glut*_, $V_{m}^{GHMT}$, $k_{cys}^{CR}$, $k_{f6p}^{PFK}$, $k_{i(glut)}^{PST}$, $V_{m}^{GLUTEX}$, $V_{m}^{GTHO}$, $V_{m}^{PSP}$, $k_{i(gap)}^{GAPDH}$, $k_{gssg}^{GTHO}$, $k_{gap}^{GAPDH}$, *dh*_2_*o*_2_, $V_{m}^{GLYex}$, $k_{i(atp)}^{GS}$, $V_{m}^{PFK}$, $k_{i(glut)}^{GLUD}$
**H2**	$k_{i(atp)}^{HK}$, $k_{oxrad}^{SOD}$
**G11**	*d*_*mlthf*_, $k_{3pg}^{PGCDH}$, *L*_*oxy*_, *d*_*php*_, $k_{glut}^{XCT}$, $k_{i(nadph)}^{NOX}$, $k_{nadph}^{NOX}$, $V_{m}^{FBA}$, $k_{glucys}^{GS}$, $k_{nadph}^{CR}$, *l*_*atp*_
**NH2**	$V_{m}^{GCL}$, $V_{m}^{O_{2}}$
**HG4**	$V_{m}^{NOX}$, *l*_*nadph*_, *d*_*in*_, $k_{O_{2}}^{NOX}$
**NG4**	*l*_*glut*_, $k_{i(atp)}^{GCL}$, *l*_*nadp*_, *d*_*oxy*_
**NHG5**	$k_{adp}^{PGK}$, $k_{i(h_{2}o_{2})}^{GTHP}$, $V_{m}^{GTHP}$, $k_{h_{2}o_{2}}^{GTHP}$, $k_{gsh}^{GTHP}$

### 3.4. Identification of Combinatorial targets for pro- and anti- oxidant therapy

We have analyzed the influence of parameters sensitive in regulating *h*_*2*_*o*_*2*_ levels during the glioma scenario using the parameters listed in G11 in Table 1. These parameters do not necessarily have a role in regulating the ROS levels directly, yet show differences in the ROS levels along with changes in *gsh/gssg* and *nadph/nadp*^*+*^ ratios when varied individually or in combination. Enzymes and metabolites associated with a few of these parameters like methyltetrahydrofolate (*mlthf*) [53, 54], NADPH Oxidase (*NOX*) [32, 55, 56], cystine-glutamate antiporter (*xCT*) [57], etc., have already been implicated in having crucial role in regulating ROS levels in the cell. A few parameters associated with enzymes which do not directly regulate the ROS and glutathione production, like Phosphoglycerate Dehydrogenase (*PGCDH*), Cystine Reductase (*CR*) and Fructose Bis-phosphate Aldolase (*FBA*) have also been identified. These parameters do not necessarily show a significant difference in the ROS levels when varied individually but show a characteristic difference when varied in combinations. These changes can be utilized for designing pro-oxidant or anti-oxidant approaches for therapeutic targeting. A few of the combinations which bring distinct changes in the *h*_*2*_*o*_*2*_ level, *gsh/gssg* and *nadph/nadp*^*+*^ ratio during glioma scenario have been listed in Table 2. $k_{nadph}^{CR}$, a parameter that has been considered in the model, which catalyzes the conversion of cystine into L-cysteine subsequently using it for glutathione production, is found to have an effect on the *h*_*2*_*o*_*2*_ level, *gsh/gssg* and *nadph/nadp*^*+*^ ratio when varied in combination with other parameters. Availability of oxygen in the ECM for cellular uptake (*L*_*oxy*_) when modulated in combination with $k_{nadph}^{CR}$ show a significant change in level. Interestingly, a combinatorial variation of *L*_*oxy*_ with $V_{m}^{FBA}$ cause a decline in the *h*_*2*_*o*_*2*_ level which can be utilized for anti-oxidant therapy, and a variation of $k_{nadph}^{CR}$ with $k_{glucys}^{GS}$ induces changes the *h*_*2*_*o*_*2*_ level which can possibly be used for pro-oxidant therapeutic design. Table 2 shows the possible utility of these combinations in pro- or anti-oxidant therapy depending upon their influence on the *h*_*2*_*o*_*2*_ level, *gsh/gssg* and *nadph/nadp*^*+*^ ratio. The changes in *h*_*2*_*o*_*2*_, *gsh/gssg* and *nadph/nadp*^*+*^ profiles due to these combinatorial variations in glioma scenario are shown in S6 Fig of S1 Text and the values for which the profiles are obtained have been listed in S3 Table of S1 Text. These combinations could be explored for their possible therapeutic abilities in the development of future therapeutic strategies. Altering the kinetic parameters like the V_max_ or k_m_ of an enzyme is challenging, though possible with the help of enzyme modulators and competitive and non-competitive inhibitors. Non-competitive inhibitors are capable of altering the V_max_ of an enzyme while keeping the k_m_ unaltered, while competitive inhibitors can alter the k_m_ of the enzyme [58]. A few of the inhibitors for the aforementioned enzymes have already been reported in literatures and have been tested in *in-vivo* experiments, which could be checked for their effectiveness in the present context. For e.g. iodoacetate, N-ethylmaleimide (NEM) and 5,5 ´-dithiobis-(2-nitrobenzoate) (DTNB) have been reported as potent inhibitors of glutathione synthase [59], and neopterin, magnolol, apocyanin and gliotoxin for NADPH oxidase [60]. However, the exact type of inhibition for these inhibitors i.e. competitive or non-competitive is not yet known and has to be understood. A context-based understanding of the involvement of these parameters in *h*_*2*_*o*_*2*_ production or scavenging mechanism under different conditions has to be made through experiments in order to employ them for therapeutic strategies.

**Table 2:** Combinatorial effect of sensitive parameters on gliomas.

Sr. No.	Parameter 1	Parameter 2	Variation of Parameter 1*	Variation of Parameter 2*	Effect
1.	$k_{i(nadph)}^{NOX}$	$k_{nadph}^{CR}$	Decrease	Increase	Pro-oxidant
2.	$k_{i(nadph)}^{NOX}$	$k_{nadph}^{NOX}$	Increase	Increase	Anti-oxidant
			Decrease	Decrease	Pro-oxidant
3.	*L*_*oxy*_	$k_{nadph}^{CR}$	Increase	Increase	Pro-oxidant
4.	$k_{i(nadph)}^{NOX}$	*l*_*atp*_	Decrease	Increase	Pro-oxidant
5.	$V_{m}^{FBA}$	*L*_*oxy*_	Decrease	Decrease	Anti-oxidant
6.	$k_{glucys}^{GS}$	$k_{nadph}^{CR}$	Decrease	Decrease	Pro-oxidant

*Increase or decrease in the parameter value with respect to normal value.

## 4. Discussion

ROS, produced as a byproduct of cellular metabolism, are often considered toxic to the cells. Nonetheless, in recent years, their functions as second messengers in signal transduction processes have been highly appreciated. In normal cells, any excess production of ROS is scavenged by the antioxidant machinery. ROS, however, exhibit a paradoxical behavior in augmenting or hindering tumor progression. It is described to have a “double-edged sword” property having both tumor-promoting and tumor-suppressing functions. Currently, both pro-oxidant and anti-oxidant approaches are employed as cancer therapeutics. However, owing to the paradoxical behavior, the employment and effectiveness of which strategy will suit a better therapeutic approach for any particular cancerous situation still remains unclear.

The oxidative status and functioning of anti-oxidant machinery have a crucial role to determine the proliferative fates of the cancer cells. The cellular *nadph/nadp*^*+*^ ratio is a measure of the reducing potential of the cells which is usually maintained high for the proper cellular functioning [24, 25]. A decline in the *nadph/nadp*^*+*^ ratio is observed in many cancer types [61, 62], although high *nadph/nadp*^*+*^ ratio was observed to promote cancer cell growth and proliferation by stimulating anabolism and by protecting cancer cells against oxidative stress during nutrient limitation [63]. Thiol ratios are another important determinant of cellular apoptotic or anti-apoptotic fates. A high *gsh/gssg* ratio is maintained under normal conditions which often changes during cancerous transformations. A decline in *gsh/gssg* ratio induces the initiation of apoptosis while an increase might help the cells escape apoptosis. The puzzling duality of ROS in exhibiting varying cellular fates is determined by a coordinated response of these factors. To understand the effect of these factors cumulatively, under different cancerous scenarios is a challenge.

Motivated by these findings, we have designed a kinetic metabolic framework for glial cell, to trace the possible changes that might be occurring within them during their transformation into gliomas. The model takes into account the metabolic reactions involved in the production of the tri-peptide complex, glutathione and ROS producing machinery. A part of the glycolytic pathway which enters the glycine-serine metabolism has been considered along with cystine and glutamate metabolism which result in the production of the components of the tri-peptide: glycine, cysteine and glutamate respectively. Herein, we considered the effect of these pathways on the anti-oxidant production machinery simultaneously looking for their effect on ROS production and scavenging and vice versa. To understand the effect of glutathione over ROS metabolism, reactions metabolizing the production of glutathione and ROS, along with the *gsh-gssg* cycle have been considered. Important reactions involving the *nadph-nadp*^*+*^ conversions have been considered, to take into account the changes in *nadph/nadp*^*+*^ ratio while manipulating *h*_*2*_*o*_*2*_-glutathione profiles. We postulate that the paradoxical behavior of ROS is governed by changes in the *nadph/nadp*^*+*^ and *gsh/gssg* when considered together. An increase in *h*_*2*_*o*_*2*_ along with a decline in both *gsh/gssg* and *nadph/nadp*^*+*^ will disrupt the cellular redox status and drive the cell towards apoptosis by inducing toxicity due to accumulation of ROS.

Numerical simulations of the model provide us with a set of sensitive enzyme parameters, which are affected during a transition from normal glial conditions to hypoxia to the development of gliomas. Upon introducing variations into the parameters which are sensitive under normal glial conditions, interesting changes in the dynamics of *h*_*2*_*o*_*2*_, *nadph/nadp*^*+*^ and *gsh/gssg* could be observed. The uptake of oxygen by the cells has been represented by the Michaelis-Menten equation form where $k_{m}^{O_{2}}$ determines the affinity of the cell for external oxygen. A decrease in $k_{m}^{O_{2}}$ results into a decline in the external oxygen concentration as the cellular affinity for external oxygen increases. This represents a condition with high cellular oxygen demand which is reflected as hypoxia in the external microenvironment.

*GTHP* is one of the important enzymes involved in the regulation of *gsh/gssg*, along with controlling the cellular content of *h*_*2*_*o*_*2*_ and maintaining *nadph/nadp*^*+*^. Through our simulation on the glial cell model, we observe that with an increase in $V_{m}^{GTHP}$ there is a considerable decline in the *h*_*2*_*o*_*2*_ level and *gsh/gssg* ratio, although the effect on *nadph/nadp*^*+*^ ratio is only trivial. *GTHO* is another important enzyme, and the *GTHP-GTHO* duo completes the *gsh*-*gssg* cycle. Not much difference in *gsh/gssg* and *nadph/nadp*^*+*^ could be observed for a change of *GTHO* alone keeping all other parameters fixed. However, simultaneously varying *GTHP* and *GTHO* result into interesting changes in *nadph/nadp*^*+*^ and *gsh/gssg* with a decrease in *h*_*2*_*o*_*2*_ level. We interpret that at any given time point under normal condition there is a decline in the *nadph/nadp*^*+*^ and *gsh/gssg* until the *gsh-gssg* cycle neutralizes *h*_*2*_*o*_*2*_ concentration to micromolar levels rendering it non-toxic to cellular processes, after which the cell regains a stable *nadph/nadp*^*+*^ and *gsh/gssg* ratio. The involvement of NADPH Oxidase (*NOX*) is found to be crucial in the present simulation scenario. The activity of *NOX* determines the rate of production of free oxygen radicals which act as a substrate for superoxide dismutase (*SOD*) and is readily converted into *h*_*2*_*o*_*2*_ given $V_{m}^{SOD}$ is sufficiently high. An increase in the *NOX* activity rapidly increases the *h*_*2*_*o*_*2*_ level decreasing both *nadph/nadp*^*+*^ and *gsh/gssg* ratio.

While analyzing the dynamics of these parameters under changing affinity for oxygen, we observe that under hypoxic conditions increase in $V_{m}^{GTHP}$ acts as the protective barrier against ROS by readily neutralizing *h*_*2*_*o*_*2*_ levels at the cost of reduced *gsh/gssg*, although the change in *nadph/nadp*^*+*^ ratio remains trivial. A decrease in the $V_{m}^{GTHP}$ can however help cancer development by causing *h*_*2*_*o*_*2*_ accumulation and inducing oncogenic signal transductions. The changes occurring due to an increase in activity of *NOX* and *GTHO* under hypoxia could also be interpreted as a condition initiating cancer development. While increasing *NOX* activity under hypoxia certainly disrupts cellular redox balance by reducing *gsh/gssg* and *nadph/nadp*^*+*^, the effect of changes in *GTHO* activity is minimal at a cost of sudden decrease in *nadph/nadp*^*+*^ and *gsh/gssg* ratios with its effect being severe at lower values. When considered together, changes in *NOX* and *GTHO* activity under normal and hypoxic conditions show substantial differences suggesting their involvement in tumor initiation.

A decrease in $V_{m}^{GTHP}$ and $V_{m}^{GTHO}$, and an increase in $V_{m}^{NOX}$ along with a low $k_{m}^{O_{2}}$ were considered to create a situation under hypoxia with multiple mutations representing a glioma-like situation. Comparisons of sensitive parameters under normal, hypoxic and glioma-like situations provide an insight into the directly and distantly related parameters which affect the production of *gsh*, *gssg* and *h*_*2*_*o*_*2*_. Through parameter variation analysis of sensitive parameters under the glioma-like scenario, it is observed that different values of $V_{m}^{GTHO}$ has differing effect on the overall redox status of the cell. We interpret that differing *GTHO* activity during cancerous transformation can govern the pro-apoptotic or anti-apoptotic fate. This partially accounts for the paradoxical behavior of ROS and helps in therapeutic determination of pro-oxidant or anti-oxidant approach either by augmenting glioma cell death and clearance or by controlling it using anti-oxidant therapies. We propose that under high *GTHO* activity an anti-oxidative approach will be suitable to control glioma progression, whereas under low *GTHO* activity a pro-oxidative approach will be appropriate to induce apoptosis of the glioma cells.

Further analysis of the glioma scenario created *in-silico* in the model shows the involvement of non-trivial parameters in the regulation of *gsh*, *gssg* and *h*_*2*_*o*_*2*_. It is interesting to note that a combinatorial parameter variation of enzymes belonging to glycolytic pathway ($V_{m}^{FBA}$) and cysteine metabolism ($k_{nadph}^{CR}$) could induce changes in the *h*_*2*_*o*_*2*_ level along with changes in *nadph/nadp*^*+*^ and *gsh/gssg* profiles during glioma. Additionally, a combinatorial variation of a few other parameters like $k_{glucys}^{GS}$,*L*_*oxy*_, *l*_*atp*_ and $k_{nadph}^{NOX}$ which are not directly involved in ROS manipulation also show changes in *h*_*2*_*o*_*2*_ level. Combinatorial responses of these parameters have been captured which suggest the possibility of utilizing these combinations in designing pro- or anti-oxidant therapeutic approaches based on their effect on ROS manipulation. The present model has been tailored for glial and glioma conditions; however, the utility of the model can be extended to simulate the dynamics of antioxidant machinery for cells under oxidative stress and to other tumor types exhibiting ROS manipulation. Since hypoxia and oxidative stress are characteristics of most tumors and antioxidant machinery plays a central role in maintaining redox balances in all cell types, this model can be used to simulate various scenarios related to redox imbalances in other types of cancers as well. Sensitive parameters for ROS manipulation under normal and cancerous conditions for different cancers can be obtained, and combinatorial targets for pro- and antioxidant therapy could be identified.

## 5. Conclusion

In the present work, we have demonstrated the effect of redox and thiol status of the cell and the antioxidants in maintaining ROS levels by considering *h*_*2*_*o*_*2*_ levels in particular, in normal glial cells and gliomas. *GTHP*, *GTHO*, and *NOX* are important in determining transition from normal glial to hypoxia to glioma situation and in regulating *h*_*2*_*o*_*2*_ levels with the cell. Additionally, changes in the redox and thiol status represented by *nadph/nadp*^*+*^ and *gsh/gssg* respectively, along with changes in the enzymes can determine the pro-apoptotic or anti-apoptotic fate of the gliomas. The differing activity of *GTHO* during glioma development helps in understanding the paradoxical behavior of *h*_*2*_*o*_*2*_ in gliomas and hence is helpful in determining the selection of therapeutic strategies: pro-oxidant or anti-oxidant, against glioma progression. Also, the involvement of enzymes which are not directly involved in the regulation of *h*_*2*_*o*_*2*_ but affect the process by inducing effect distantly in the metabolic network are important in augmenting the effectiveness of the selected therapeutic approach. The effect of hypoxia in the model has been evaluated by the affinity of the cell for oxygen uptake. Further inclusion of the impact of endogenous factors like *HIF1α*, the faulty oxidative phosphorylation, etc., inducing hypoxia can be insightful and will be considered in the future extension of the work. The understanding of these mechanisms and the identification of important enzymes that affect the ROS manipulation process can potentially build a better future prospect of developing effective and efficient therapeutic strategies for the treatment of gliomas.

## Supporting information

S1 Text Model parameters. The Supplementary Text contains the description of the equations, parameters and state variables used for the model simulations (Section 1, 2 and 4 and S1 and S2 Tables). S3 Table contains the values of parameters for which pro- and anti-oxidant effects are obtained in the glioma scenario. The text also contains 6 figures (S1-S6 Figs), descriptions of which are provided below.
(PDF)
