# Supplementary material for: Differential suitability of reactive oxygen species and the role of glutathione in regulating paradoxical behavior in gliomas: A mathematical perspective
Source: PLoS One. 2020 Jun 25;15(6):e0235204. doi: 10.1371/journal.pone.0235204 (PMC7316271; doi:10.1371/journal.pone.0235204)
Supplement: S1 Text — The Supplementary Text contains the description of the equations, parameters and state variables used for the model simulations (Section 1, 2 and 4 and S1 and S2 Tables). S3 Table contains the values of parameters for which pro- and anti-oxidant effects are obtained in the glioma scenario. The text also contains 6 figures (S1-S6 Figs), descriptions of which are provided below. (PDF) [file pone.0235204.s001.pdf]

Abbreviations: *GLCT*: Glucose transporter; *HK*: Hexokinase; *PGI*: Phosphoglucose isomerase; *PFK*: Phosphofructokinase; *FBA*: Fructose-1,6-bisphosphate aldolase; *TPI*: Triose phosphate isomerase; *GAPDH*: Glyceraldehyde-3-phosphate dehydrogenase; *PGK*: Phosphoglycerate kinase; *G6PDH*: Glucose-6-phosphate dehydrogenase; *GLUTEX*: Glutamate exchanger; *GLUD*: glutamate dehydrogenase; *GCL*: Glutamate-cysteine ligase; *GS*: Glutathione synthetase; *GLYex*: Glycine exchanger; *PGCDH*: Phosphoglycerate dehydrogenase; *PSP*: Phosphoserine phosphatase; *PST*: Phosphoserine transaminase; *GHMT*: Glycine hydroxymethyltransferase; *XCT*: Cystine-glutamate antiporter; *CR*: Cystine reductase; *GTHP*: Glutathione peroxidase; *GTHO*: Glutathione oxidoreductase; *NOX*: NADPH oxidase; *SOD*: Superoxide dismutase; ROS: Reactive oxygen species.

Nomenclature: All the enzymes names have been abbreviated in the block and italicized. For e.g. *PGK* refers to the enzyme phosphoglycerate kinase. All the metabolites have been abbreviated in small and italicized. For e.g. *gly* refers to the metabolite glycine. Only two compartments have been used in the model: extracellular matrix (e) and cytosol (c). To represent the compartment in which the metabolite belongs, the compartment abbreviation is used as a subscript. For e.g. glc_e refers to the metabolite glucose in the extracellular matrix.

1. Rate Equations in Cleland Nomenclature

Kinetic equations for all the metabolic reactions were written following the Cleland nomenclature for mono-substrate, bi-substrate, and ter-substrate reactions. Kinetic rate equations for the enzymes have been previously derived mechanistically and reported in various literature. Initial kinetics with first-order reaction rates of the enzymatic reactions have been considered for writing the equations. Since initial rates are usually measured in the presence of substrates only, there are no product terms present in the equations. Values for all the parameters have been listed in Table S1 and initial values of all the variables have been provided in section 4 of the supporting information.

Central Carbon Metabolism

Glycolysis: Kinetic rate equations for the enzymes belonging to the first half of glycolysis, from where it branches to the glycine-serine pathway have been considered in the model. The kinetics is mostly represented by uni-uni and bi-bi mechanisms. The equation forms for most of the reactions are similar except for *GAPDH* and *PGK*. Competitive inhibition of *GAPDH* by h_2o_2 has been considered. The rate kinetics of *PGK* follows a partial rapid equilibrium random bi-bi steady-state kinetics [1] where the binding of *1,3bpg* with the enzyme is transient and has not been considered in the denominator.

Eq.1:

$$v_{GLCT} = \frac{V_m^{GLCT}.glc_e}{k_{glc_e}^{GLCT} + glc_e} \; [Uni\ Uni\ Michaelis\ Menten]$$

Eq.2:

$$v_{HK} = \frac{V_m^{HK}.(atp.glc_c)}{(k_{i(atp)}^{HK}.k_{glc_c}^{HK} + k_{glc_c}^{HK}.atp + k_{atp}^{HK}.glc_c + atp.glc_c)} \begin{bmatrix} Rapid\ Equilibrium\ Random \\ Bi - Bi\ mechanism \end{bmatrix}$$

Eq.3:

$$v_{PGI} = \frac{V_m^{PGI}.g6p}{k_{g6p}^{PGI} + g6p} \begin{bmatrix} Steady\ state\ uni - uni\ mechanism; \\ Briggs\ Haldane\ form\ of\ Michealis\ Menten\ kinetics \end{bmatrix}$$

Eq.4:

$$v_{PFK} = \frac{V_m^{PFK}(atp.f6p)}{(k_{i(atp)}^{PFK}.k_{f6p}^{PFK} + k_{f6p}^{PFK}.atp + k_{atp}^{PFK}.f6p + atp.f6p)} \begin{bmatrix} Rapid\ Equilibrium \\ Random\ Bisubstrate \end{bmatrix}$$

Eq.5:

$$v_{FBA} = \frac{V_m^{FBA}.f16bp}{k_{f16bp}^{FBA} + f16bp} \; [Ordered\ Uni\ Bi\ steady\ state]$$

Eq.6:

$$v_{TPI} = \frac{V_m^{TPI}.dhap}{k_{dhap}^{TPI} + dhap} \; [Simple\ steady\ state\ Uni - Uni\ Mechanism]$$

Eq.7:

$$v_{GAPDH} = \frac{V_m^{GAPDH}.(gap.nad)}{[k_{i(gap)}^{GAPDH}.k_{nad}^{GAPDH} + k_{gap}^{GAPDH}.nad\ .(1+\frac{h_2o_2}{k_{ii(h_2o_2)}^{GAPDH}}) + k_{nad}^{GAPDH}.gap + gap.nad]} \begin{bmatrix} Ordered\ bi\ bi \\ Steady\ State\ kinetics; \\ h2o2\ inhibition \end{bmatrix}$$

Eq.8:

$$v_{PGK} = \frac{V_m^{PGK}.(1,3bpg.adp)}{(k_{i(1,3bpg)}^{PGK}.k_{adp}^{PGK} + k_{adp}^{PGK}.1,3bpg + 1,3bpg.adp)} \begin{bmatrix} Partial\ Rapid \\ Equilibrium\ Random \\ Bi - Bi\ mechanism \end{bmatrix}$$

Pentose Phosphate Pathway: The pentose phosphate pathway has been represented by considering the enzymatic reaction G6PDH. The assumption here is that the final products of the pathway, i.e., fructose and glyceraldehyde 3-phosphate re-enter the glycolytic pathway. The whole pathway has been reduced to a single equation and the parameter values for the equation have been determined by parameter estimation technique to represent the real biological situation. Competitive inhibition of the pathway by atp has been considered.

Eq.9:

$$v_{G6PDH} = \frac{V_m^{G6PDH}(g6p.nadp)}{[(k_{i(g6p)}^{G6PDH}.k_{nadp}^{G6PDH} + k_{g6p}^{G6PDH}.nadp).(1+\frac{atp}{k_{ii(atp)}^{G6PDH}}) + k_{nadp}^{G6PDH}.g6p + g6p.nadp]} \begin{bmatrix} Ordered\ Bi\ Bi\ Mechanism \\ with\ Competitive\ inhibition \\ of\ \ g6p\ by\ atp_c \end{bmatrix}$$

Amino Acid Metabolism

Glutamate Metabolism: A part of the glutamate metabolism has been considered which includes the transport of glutamate from the extracellular matrix to the cytoplasm where a part of it is utilized in the glutathione metabolism and a part is converted into akg production by the activity of glutamate dehydrogenase and the rest is utilized in other cellular processes.

Eq.10

$$v_{GLUTEX} = \frac{V_m^{GLUTEX}.glut_e}{(k_m^{GLUTEX} + glut_e)} [Uptake\ of\ glutamate\ from\ extracellular\ matrix]$$

Eq.11:

$$v_{GLUD} = \frac{V_m^{GLUD}.(glut.nadp)}{(k_{i(glut)}^{GLUD}.k_{nadp}^{GLUD} + k_{nadp}^{GLUD}.glut + k_{glut}^{GLUD}.nadp + glut.nadp)} \ [Ordered\ Bi\ Bi]$$

Glycine Serine Metabolism: All the enzymatic reactions involved in the production of serine from 3pg and conversion of serine to glycine have been considered. Along with it, the exchange of glycine from the extracellular matrix has also been considered.

Eq.12:

$$v_{GLYex} = \frac{V_m^{GLYex}.gly_e}{k_m^{GLYex} + gly_e} [Michaelis\ Menten]$$

Eq.13

$$v_{PGCDH} = \frac{V_m^{PGCDH}.(3pg.nad)}{(k_{i(3pg)}^{PGCDH}.k_{nad}^{PGCDH} + k_{nad}^{PGCDH}.3pg + k_{3pg}^{PGCDH}.nad + 3pg.nad)} \ [Ordered\ Bi\ Bi]$$

Eq.14:

$$v_{PSP} = \frac{V_m^{PSP}.pser}{(k_m^{PSP} + pser)}$$

Eq.15:

$$v_{GHMT} = \frac{V_m^{GHMT}.(ser.thf)}{(k_{i(ser)}^{GHMT}.k_{thf}^{GHMT} + k_{thf}^{GHMT}.ser + k_{ser}^{GHMT}.thf + ser.thf)} \ [Ordered\ Bi\ Bi]$$

Eq.16:

$$v_{PST} = \frac{V_m^{PST}.(glut.3php)}{(k_{i(glut)}^{PST}.k_{3php}^{PST} + k_{3php}^{PST}.glut + k_{glut}^{PST}.3php + glut.3php)} \quad \begin{bmatrix} Rapid\ Equilibrium \\ Random\ Bi\ Bi \end{bmatrix}$$

Cysteine metabolism: This considers the transport of cystine from the extracellular matrix via the glutamate-cystine antiporter (*xCT*) and the conversion of cystine into cysteine, which is used as a component for building the tripeptide complex of glutathione.

Eq.17:

$$v_{XCT} = \frac{V_m^{XCT}.(cys_e.glut)}{(k_{i(cys_e)}^{XCT}.k_{glut}^{XCT} + k_{glut}^{XCT}.cys_e + k_{cys_e}^{XCT}.glut + cys_e.glut)}$$

Eq.18:

$$v_{CR} = \frac{V_m^{CR}.(cys.nadph)}{(k_{i(cys)}^{CR}.k_{nadph}^{CR} + k_{nadph}^{CR}.cys + k_{cys}^{CR}.nadph + cys.nadph)} \quad [Ordered\ Bi\ Bi]$$

Thiol Metabolism

Glutathione Metabolism: This pathway includes the involvement of two important enzymes, glutamyl-cysteine ligase and glutathione synthase. The former catalyzes the formation of glutamyl-cysteine which is converted into the tripeptide complex of glutathione in the later reaction. Both the reactions involve the utilization of *atp* for the conversion and hence represented by three metabolites ordered ter-bi equation form.

Eq.19:

$$v_{GCL} = \frac{V_m^{GCL}.(atp.glut.cysL)}{(k_{i(atp)}^{GCL}.k_{i(glut)}^{GCL}.k_{cysL}^{GCL} + k_{i(glut)}^{GCL}.k_{cysL}^{GCL}.atp + k_{i(atp)}^{GCL}.k_{glut}^{GCL}.cysL + k_{cysL}^{GCL}.atp.glut + k_{glut}^{GCL}.atp.cysL + k_{atp}^{GCL}.glut.cysL + atp.glut.cysL)} \quad [Ordered\ Ter\ Bi]$$

Eq.20:

$$v_{GS} = \frac{V_m^{GS}.(atp.gly.glucys)}{(k_{i(atp)}^{GS}.k_{i(gly)}^{GS}.k_{glucys}^{GS} + k_{i(gly)}^{GS}.k_{glucys}^{GS}.atp + k_{i(atp)}^{GS}.k_{gly}^{GS}.glucys + k_{glucys}^{GS}.atp.gly + k_{gly}^{GS}.atp.glucys + k_{atp}^{GS}.gly.glucys + atp.gly.glucys)}$$

GSH-GSSG cycle: This is represented by two enzymes, glutathione peroxidase which catalyzes the conversion of gsh into $gssg$ while neutralizing h_2o_2 to h_2o and glutathione oxidoreductase which replenishes gsh back into the system by converting $gssg$ to gsh with the involvement of $nadph$. The equations for the reactions have been represented using an ordered bi-bi mechanism.

Eq.21:

$$v_{GTHP} = \frac{V_m^{GTHP}.h_2o_2.gsh}{(k_{i(h_2o_2)}^{GTHP}.k_{gsh}^{GTHP} + k_{gsh}^{GTHP}.h_2o_2 + k_{h_2o_2}^{GTHP}.gsh + h_2o_2.gsh)} [Ordered\ Bi\ Uni]$$

Eq.22:

$$v_{GTHO} = \frac{V_m^{GTHO}.(gssg.nadph)}{(k_{i(gssg)}^{GTHO}.k_{nadph}^{GTHO} + k_{nadph}^{GTHO}.gssg + k_{gssg}^{GTHO}.nadph + gssg.nadph)} \quad [Ordered\ Bi\ Bi]$$

h_2o_2 production and metabolism

***h_2o_2* production machinery:** This is represented by two enzymatic reactions: NADPH oxidase (*NOX*) which converts oxygen into oxygen free radicals and superoxide dismutase (*SOD*) which converts the free radicals into h_2o_2. The former equation has been represented by ordered bi-bi mechanism and the later by a uni-uni mechanism.

Eq.23:

$$v_{NOX} = \frac{V_m^{NOX}.(nadph.O_2)}{(k_{i(nadph)}^{NOX}.k_{O_2}^{NOX} + k_{O_2}^{NOX}.nadph + k_{nadph}^{NOX}.O_2 + nadph.O_2)} \quad [Ordered\ Bi\,Bi]$$

Eq.24:

$$v_{SOD} = \frac{V_m^{SOD}.(oxrad)}{(k_{oxrad}^{SOD} + oxrad)} [Ordered\ Uni\ Bi]$$

Oxygen Uptake: The uptake of oxygen has been represented using a simple Michaelis-Menten equation form. Here V_{max} represents the maximum rate of uptake of oxygen by the cell and k_m represents affinity of the cell for external oxygen. The lower the value of k_m, the higher is the affinity for external oxygen.

Eq.25:

$$v_{O_2} = \frac{V_m^{O_2}.O_{2(ex)}}{k_m^{O_2} + O_{2(ex)}}$$

2. Differential Equations:

All the differential equations with metabolites as the variables have been formulated by considering the involvement of the metabolites in different enzymatic reactions. A few natural productions and decay rates have been considered to take into account the formation of metabolites from other sources and their utilization in other cellular processes respectively. Values to all the parameters considered in the differential equations have been tabulated in Table S1.

1. $\frac{d(glc_e)}{dt} = l_g - v_{GLCT} - d_g[glc_e]$
2. $\frac{d(glc_c)}{dt} = v_{GLCT} - v_{HK}$
3. $\frac{d(g6p)}{dt} = v_{HK} - v_{PGI} - v_{G6PDH}$

4. $$\frac{d(f6p)}{dt} = v_{PGI} + v_{G6PDH} - v_{PFK}$$

5. $$\frac{d(f16bp)}{dt} = v_{PFK} - v_{FBA}$$

6. $$\frac{d(gap)}{dt} = v_{FBA} + v_{G6PDH} + v_{TPI} - v_{GAPDH}$$

7. $$\frac{d(dhap)}{dt} = v_{FBA} - v_{TPI}$$

8. $$\frac{d(1,3bpg)}{dt} = v_{GAPDH} - v_{PGK}$$

9. $$\frac{d(3pg)}{dt} = v_{PGK} - v_{PGCDH} - d_{3pg}[3pg]$$

10. $$\frac{d(atp)}{dt} = l_{atp} + v_{PGK} - v_{HK} - v_{PFK} - v_{GCL} - v_{GS} - d_{atp}[atp]$$

11. $$\frac{d(adp)}{dt} = l_{adp} + v_{HK} + v_{PFK} + v_{GCL} + v_{GS} - v_{PGK} - d_{adp}[adp]$$

12. $$\frac{d(nadph)}{dt} = l_{nadph} + v_{G6PDH} + v_{GLUD} - v_{GTHO} - v_{NOX} - v_{CR} - d_{nadph}[nadph]$$

13. $$\frac{d(nadp)}{dt} = l_{nadp} + v_{GTHO} + v_{NOX} + v_{CR} - v_{GLUD} - v_{G6PDH} - d_{nadp}[nadp]$$

14. $$\frac{d(nadh)}{dt} = l_{nadh} + v_{GAPDH} + v_{PGCDH} - d_{nadh}[nadh]$$

15. $$\frac{d(nad)}{dt} = l_{nad} - v_{GAPDH} - v_{PGCDH} - d_{nad}[nad]$$

16. $$\frac{d(3php)}{dt} = v_{PGCDH} - v_{PST}$$

17. $$\frac{d(glut)}{dt} = v_{GLUTEX} - v_{GCL} - v_{PST} - v_{XCT} - v_{GLUD}$$

18. $$\frac{d(akg)}{dt} = v_{PST} + v_{GLUD} - d_{akg}[akg]$$

19. $$\frac{d(pser)}{dt} = v_{PST} - v_{PSP}$$

20. $$\frac{d(ser)}{dt} = v_{PSP} - v_{GHMT}$$

21. $$\frac{d(thf)}{dt} = l_{thf} - v_{GHMT} - d_{thf}[thf]$$

22. $$\frac{d(mlthf)}{dt} = l_{mlthf} + v_{GHMT} - d_{mlthf}[mlthf]$$

23. $$\frac{d(gly)}{dt} = v_{GLYex} + v_{GHMT} - v_{GS} - d_{gly}[gly]$$

24. $$\frac{d(cysL)}{dt} = v_{CR} - v_{GCL}$$

25. $$\frac{d(glucys)}{dt} = v_{GCL} - v_{GS} - d_{glucyc}[glucys]$$

26. $$\frac{d(gsh)}{dt} = v_{GS} + v_{GTHO} - v_{GTHP} - d_{gsh}[gsh]$$

27. $$\frac{d(cys)}{dt} = v_{XCT} - v_{CR}$$

28. $$\frac{d(h_2o_2)}{dt} = v_{SOD} - v_{GTHP} - d_{h_2o_2}[h_2o_2]$$

29. $$\frac{d(gssg)}{dt} = v_{GTHP} - v_{GTHO} - d_{gssg}[gssg]$$

30. $$\frac{d(glut_e)}{dt} = l_{gl} + v_{XCT} - v_{GLUTEX} - d_{gl}[glut_e]$$

31. $$\frac{d(cys_e)}{dt} = l_{cy} - v_{XCT} - d_{cy}[cys_e]$$

32. $$\frac{d(O_{2(ex)})}{dt} = L_{oxy} - v_{O_2} - d_{oxy}[O_{2(ex)}]$$

33. $$\frac{d(O_2)}{dt} = v_{O_2} + v_{SOD} - v_{NOX} - d_{in}[O_2]$$

34. $$\frac{d(oxrad)}{dt} = v_{NOX} - v_{SOD}$$

35. $\dfrac{d(gly_e)}{dt} = l_{gly(e)} - v_{GLYex} - d_{gly(e)}[gly_e]$

3. Positivity and Boundedness

Positivity:

Proposition 1: All the solutions of Eq. 1-35 (Section 2) which initiate in R_+^{35} lie in a positive solution space.

Proof: The system of equations described in Supplementary Section 2 (Eq. 1-35) can be analyzed with the initial conditions (Supplementary Section 5) defined in the thirty-five-dimensional variable space

$$\begin{aligned} R_+^{35} = [(& glc_e, glc_c, g6p, f6p, f16bp, gap, dhap, 1{,}3bpg, 3pg, atp, adp, nadph, nadp, nadh, nad, \\ & 3php, glut, akg, pser, ser, thf, mlthf, gly, cysL, glucys, gsh, cys, h_2o_2, gssg, glut_e, cys_e, O_{2(ex)}, \\ & oxrad, gly_e) \in \Re^{35} \mid (glc_e, glc_c, g6p, f6p, f16bp, gap, dhap, 1{,}3bpg, 3pg, atp, adp, nadph, \\ & nadp, nadh, nad, 3php, glut, akg, pser, ser, thf, mlthf, gly, cysL, glucys, gsh, cys, h_2o_2, gssg, \\ & glut_e, cys_e, O_{2(ex)}, oxrad, gly_e) \geq 0] \end{aligned}$$

It can be proven, that all solutions of the system in R_+^{35} remain in R_+^{35}. Hence, R_+^{35} is positively invariant, and it is sufficient to consider solutions only in R_+^{35}. In this region, the usual existence, uniqueness and continuation results hold for the system. From our numerical simulations, we have observed the existence of positive solutions for the parameter values presented in Table S1. The steady-state attained by all the variables of the model is a part of this positive solution space.

We observe that the right-hand side of Eq. 1-35 (Section 2) are smooth functions of the variables

$$\begin{aligned} (& glc_e, glc_c, g6p, f6p, f16bp, gap, dhap, 1{,}3bpg, 3pg, atp, adp, nadph, nadp, nadh, nad, 3php, glut, \\ & akg, pser, ser, thf, mlthf, gly, cysL, glucys, gsh, cys, h_2o_2, gssg, glut_e, cys_e, O_{2(ex)}, oxrad, gly_e) \end{aligned}$$

Since all the parameters are non-negative, local existence and uniqueness properties hold in R_+^{35}, and if the following necessary conditions are satisfied,

1. $l_g > d_g[glc_e]$
2. $l_{atp} > d_{atp}[atp]$
3. $l_{adp} > d_{adp}[adp]$
4. $l_{nadph} > d_{nadph}[nadph]$
5. $l_{nadp} > d_{nadp}[nadp]$
6. $l_{nadh} > d_{nadh}[nadh]$

7. $l_{nad} > d_{nad}[nad]$
8. $l_{thf} > d_{thf}[thf]$
9. $l_{mlthf} > d_{mlthf}[mlthf]$
10. $v_{SOD} > d_{h_2o_2}[h_2o_2]$
11. $l_{gl} > d_{gl}[glut_e]$
12. $l_{cy} > d_{cy}[cys_e]$
13. $L_{oxy} > d_{oxy}[O_{2(ex)}]$
14. $l_{gly(e)} > d_{gly(e)}[gly_e]$

Boundedness:

Theorem: All the solutions of Eq. 1-35 (Section 2) which initiate in R_+^{35} are uniformly bounded.

Proof:

Let

$$W_1 = [glc_e]+[3pg]+[atp]+[adp]+[nadph]+[nadp]+[nadh]+[nad]+[akg]+[thf]+[mlthf]+ [gly]+[glucys]+[gsh]+[h_2o_2]+[gssg]+[glut_e]+[cys_e]+[O_{2(ex)}]+[O_2]+[gly_e]$$

Then taking time derivative and using the equation, we have

$$\frac{dW_1}{dt} \le l_{wl} + v_{G6PDH} + v_{FBA} + v_{SOD} - v_{GCL} - v_{GS} - v_{GTHP} - d_{wl}W_1$$

where,

$$d_{wl} = \min\{d_g, d_{3pg}, d_{atp}, d_{adp}, d_{nadph}, d_{nadp}, d_{nadh}, d_{nad}, d_{akg}, d_{thf}, d_{mlthf}, d_{gly}, d_{glucys}, d_{gsh}, d_{h_2o_2}, d_{gssg}, d_{gl}, d_{cy}, d_{oxy}, d_{in}, d_{gly(e)}\}$$

and $l_{wl} = \max\{l_g, l_{atp}, l_{adp}, l_{nadph}, l_{nadp}, l_{nadh}, l_{nad}, l_{thf}, l_{mlthf}, l_{gl}, l_{cy}, L_{oxy}, l_{gly(e)}\}$

Let $L_p = V_m^{G6PDH} + V_m^{FBA} + V_m^{SOD}$ and $L_n = V_m^{GCL} + V_m^{GS} + V_m^{GTHP}$ where $v_{G6PDH} + v_{FBA} + v_{SOD} \le L_p$ since $v_{G6PDH} \le V_m^{G6PDH}$ and so on; and $v_{GCL} + v_{GS} + v_{GTHP} \le L_n$ since $v_{GCL} \le V_m^{GCL}$ and so on.

$$\Rightarrow \frac{dW_1}{dt} \le l_{wl} + L_p - d_{wl}W_1$$

$$\Rightarrow \frac{dW_1}{dt} + d_{wl}W_1 \le l_{wl} + L_p$$

$$\Rightarrow \frac{dW_1}{dt} + d_{wl}W_1 \le \theta_1 \text{ where, } \theta_1 = l_{wl} + L_p$$

From the theory of differential inequalities, we then obtain

$$0 < W_1([glc_e],[3pg],[atp],[adp],[nadph],[nadp],[nadh],[nad],[akg],[thf],[mlthf],[gly],[glucys],$$
$$[gsh],[h_2o_2],[gssg],[glut_e],[cys_e],[O_{2(ex)}],[O_2],[gly_e]) < \frac{\theta_1}{d_{wl}}(1-e^{-d_{wl}t}) + W_1([glc_e](0),[3pg](0),$$
$$[atp](0),[adp](0),[nadph](0),[nadp](0),[nadh](0),[nad](0),[akg](0),[thf](0),[mlthf](0),[gly](0),$$
$$[glucys](0),[gsh](0),[h_2o_2](0),[gssg](0),[glut_e](0),[cys_e](0),[O_{2(ex)}](0),[O_2](0),[gly_e](0))e^{-d_{wl}t}$$

and for $t \to \infty$, it follows $0 < W_1 < \frac{\theta_1}{d_{wl}}$.

Hence all solutions of $W_1(t)$ that initiate at $W_1(0) \in \mathrm{R}^{35}$ are confined to the region: $G_1 = \{(W_1)^T \in \mathrm{R}^{35} : W_1 = \frac{\theta_1}{d_{wl}} + \varepsilon_1$ for any $\varepsilon_1 > 0\}$ for all $t \ge T^*$ where T^* depends on the initial value $(W_1(0))^T$ and lie in a positive solution space provided the necessary conditions specified in Proposition 1 are satisfied.

4. Parameter values and reported range of initial values of variables:

All the parameters have been defined and the values have been provided along with the reference to the literature from which they have been obtained. A set of parameters has been estimated using the Delayed Rejection Adaptive Metropolis (DRAM) algorithm of Markov Chain Monte Carlo (MCMC) Toolbox in MATLAB 2017a. These parameters have been referenced as “Estimated”. Another set of parameters has been referenced as “Expected”. These parameters have been estimated by varying the parameters within the biologically feasible ranges as reported in various literature to determine their expected value to calibrate the model with experimental observations.

Table S1: Parameter values and their units used for the model simulation and their references

Sr. No.	Parameter	Description	Value	Unit	Reference
1.	V_m^{GLCT}	V_{max} of Glucose transporter	7.67	$mM\ hr^{-1}$	[1]
2.	$k_{glc_e}^{GLCT}$	Rate constant for association of cytosolic glucose with Glucose transporter	2.1	mM	[1]
3.	l_g	Rate of glucose influx from other extracellular sources in the ECM	5.6	$mM\ hr^{-1}$	Expected*
4.	d_g	Depletion of extracellular glucose for utilization by other cell types	0.2	hr^{-1}	
5.	V_m^{HK}	V_{max} of Hexokinase	$9.59*10^2$	$mM\ hr^{-1}$	[1]
6.	$k_{i(atp)}^{HK}$	Rate constant for dissociation of ATP with Hexokinase	1	mM	[1]
7.	$k_{glc_c}^{HK}$	Rate constant for association of cytosolic glucose with Hexokinase	0.47	mM	**
8.	k_{atp}^{HK}	Rate constant for association of ATP with Hexokinase	1	mM	[1]
9.	V_m^{PGI}	V_{max} of Phosphoglucose Isomerase	$2.4*10^3$	$mM\ hr^{-1}$	[1]
10.	k_{g6p}^{PGI}	Rate constant for association of G6P with Phosphoglucose Isomarase	$9.6*10^{-1}$	mM	[1]
11.	V_m^{PFK}	V_{max} of Phosphofructokinase	$2.63*10^2$	$mM\ hr^{-1}$	[1]
12.	k_{f6p}^{PFK}	Rate constant for association of F6P with Phosphofructokinase	$6*10^{-2}$	mM	[1]
13.	k_{atp}^{PFK}	Rate constant for association of ATP with Phosphofructokinase	$6.8*10^{-2}$	mM	[1]
14.	$k_{i(atp)}^{PFK}$	Rate constant for dissociation of ATP from Phosphofructokinase	10.204	mM	Estimated
15.	V_m^{FBA}	V_{max} of Fructose Bisphosphate Aldolase	$1.33*10^2$	$mM\ hr^{-1}$	[1]
16.	k_{f16bp}^{FBA}	Rate constant for association of F16BP with Fructose Bisphosphate Aldolase	$5*10^{-2}$	mM	[1]
17.	V_m^{TPI}	V_{max} of Triosephosphate Isomerase	$5.10*10^2$	$mM\ hr^{-1}$	[1]
18.	k_{dhap}^{TPI}	Rate constant for association of Triosephosphate Isomerase	$1.62*10^{-1}$	mM	[1]
19.	V_m^{GAPDH}	V_{max} of Glyceraldehyde phosphate dehydrogenase	781	$mM\ hr^{-1}$	[2]
20.	k_{gap}^{GAPDH}	Rate constant for association of GAP with Glyceraldehyde phosphate dehydrogenase	1.4	mM	[3]

21.	$k_{i(gap)}^{GAPDH}$	Rate constant for dissociation of GAP from Glyceraldehyde phosphate dehydrogenase	$1.59*10^{-16}$	mM	[1]
22.	$k_{ii(h_2o_2)}^{GAPDH}$	Inhibition constant of H2O2 for Glyceraldehyde phosphate dehydrogenase	21.044	mM	Estimated
23.	k_{nad}^{GAPDH}	Rate constant for association of NAD with Glyceraldehyde phosphate dehydrogenase	1.3	mM	[3]
24.	l_{nad}	Rate of formation of intracellular NAD^+ from other internal sources	12.5	$mM\ hr^{-1}$	Expected
25.	d_{nad}	Depletion of NAD^+ in other intracellular reactions	0.5	hr^{-1}	
26.	V_m^{PGK}	V_{max} of Phosphoglycerate kinase	$2.21*10^2$	$mM\ hr^{-1}$	[2]
27.	$k_{i(1,3bpg)}^{PGK}$	Rate constant for dissociation of BPG from Phosphoglycerate kinase	1.6	mM	[1]
28.	k_{adp}^{PGK}	Rate constant for association of ADP with Phosphoglycerate kinase	0.1	mM	[1]
29.	d_{pg}	Natural decay of intracellular 3-phosphoglycerate	1.8	hr^{-1}	Expected
30.	V_m^{PGCDH}	V_{max} of Phosphoglycerate dehydrogenase	0.0021	$mM\ hr^{-1}$	[4]
31.	k_{nad}^{PGCDH}	Rate constant for association of NAD with Phosphoglycerate kinase	0.022	mM	[5]
32.	k_{3pg}^{PGCDH}	Rate constant for association of PG with Phosphoglycerate kinase	0.26	mM	[5]
33.	$k_{i(3pg)}^{PGCDH}$	Rate constant for dissociation of PG from Phosphoglycerate kinase	20	mM	Estimated
34.	d_{php}	Natural decay of intracellular phosphohydroxy phosphate	0.005	hr^{-1}	Expected
35.	V_m^{PST}	V_{max} of Phosphoserine transaminase	0.081	$mM\ hr^{-1}$	[6]
36.	$k_{i(glut)}^{PST}$	Rate constant for dissociation of Glutamate from Phosphoserine transaminase	7.42	mM	[6]
37.	k_{3php}^{PST}	Rate constant for association of PHP from Phosphoserine transaminase	0.005	mM	[6]
38.	k_{glut}^{PST}	Rate constant for association of Glutamate with Phosphoserine transaminase	1.2	mM	[6]
39.	d_{akg}	Utilization of α-ketoglutarate into intracellular reactions	0.8	hr^{-1}	Expected
40.	V_m^{PSP}	V_{max} of Phosphoserine Phosphatase	0.162	$mM\ hr^{-1}$	[7]

41.	k_m^{PSP}	K_m of Phosphoserine transaminase	0.1	mM	[8]
42.	V_m^{GHMT}	V_{max} of Glycine hydroxymethyltransferase	40	$mM\ hr^{-1}$	[9]
43.	k_{thf}^{GHMT}	Rate constant for association of THF with Glycine hydroxymethyltransferase	0.05	mM	[9, 10]
44.	k_{ser}^{GHMT}	Rate constant for association of Serine with Glycine hydroxymethyltransferase	0.6	mM	[9, 10]
45.	$k_{i(ser)}^{GHMT}$	Rate constant for dissociation of Serine from Glycine hydroxymethyltransferase	14	mM	Estimated
46.	V_m^{GLUTEX}	V_{max} of Glutamate exchanger	38.691	$mM\ hr^{-1}$	Estimated
47.	k_m^{GLUTEX}	K_m of Glutamate exchanger	0.097371	mM	Estimated
48.	l_{thf}	Rate of formation of tetrahydofolate from internal reactions	4	$mM\ hr^{-1}$	Expected***
49.	d_{thf}	Depletion of tetrahydrofolate in other intracellular reactions	0.5	hr^{-1}	Expected
50.	l_{mlthf}	Rate of formation of methyl-tetrahydofolate from other internal reactions	6	$mM\ hr^{-1}$	Expected***
51.	d_{mlthf}	Depletion of methyl- tetrahydrofolate in intracellular reactions	0.5	hr^{-1}	Expected
52.	V_m^{GLUD}	V_{max} of Glutamate dehydrogenase	$5.55*10^3$	$mM\ hr^{-1}$	[1]
53.	$k_{i(glut)}^{GLUD}$	Rate constant for dissociation of Glutamate from Glutamate dehydrogenase	3.5	mM	[1]
54.	k_{glut}^{GLUD}	Rate constant for association of Glutamate with Glutamate dehydrogenase	3.5	mM	[1]
55.	k_{nadp}^{GLUD}	Rate constant for association of NADP with Glutamate dehydrogenase	5.6	mM	Estimated
56.	l_{glut}	Rate of glutamate influx into the ECM from other cellular processes	3	$mM\ hr^{-1}$	Expected
57.	d_{glut}	Depletion of extracellular glutamate from other cellular processes	0.3	hr^{-1}	
58.	V_m^{GCL}	V_{max} of Glutamyl-cysteine Ligase	14	$mM\ hr^{-1}$	Estimated
59.	k_{cysL}^{GCL}	Rate constant for association of Cysteine with Glutamyl-cysteine Ligase	0.07	mM	[11]
60.	k_{glut}^{GCL}	Rate constant for association of Glutamate with Glutamyl-cysteine Ligase	0.46	mM	[11]
61.	k_{atp}^{GCL}	Rate constant for association of ATP with Glutamyl-cysteine Ligase	0.44	mM	[11]

62.	$k_{i(atp)}^{GCL}$	Rate constant for dissociation of ATP from Glutamyl-cysteine Ligase	9.73	mM	Estimated
63.	$k_{i(glut)}^{GCL}$	Rate constant for dissociation of Glutamate from Glutamyl-cysteine Ligase	9.72	mM	Estimated
64.	d_{glucys}	Natural decay of intracellular gamma-glutamyl cysteine	0.08	hr^{-1}	Expected
65.	V_m^{GS}	V_{max} of Glutathione synthase	0.1174	$mM\ hr^{-1}$	[12]#
66.	k_{glucys}^{GS}	Rate constant for association of Glutamyl-cysteine with Glutathione synthase	0.99	mM	[13]
67.	k_{gly}^{GS}	Rate constant for association of Glycine with Glutathione synthase	1.37	mM	[13]
68.	k_{atp}^{GS}	Rate constant for association of ATP with Glutathione synthase	0.23	mM	[13]
69.	$k_{i(atp)}^{GS}$	Rate constant for dissociation of ATP from Glutathione Synthase	11.8	mM	Estimated
70.	$k_{i(gly)}^{GS}$	Rate constant for dissociation of Glycine from Glutathione Synthase	6.0699	mM	Estimated
71.	V_m^{NOX}	V_{max} of NADPH Oxidase	0.0468	$mM\ hr^{-1}$	[14]
72.	$k_{O_2}^{NOX}$	Rate constant for association of Oxygen with NADPH Oxidase	0.22	mM	[15]
73.	k_{nadph}^{NOX}	Rate constant for association of NADPH with NADPH Oxidase	0.055	mM	[15]
74.	$k_{i(nadph)}^{NOX}$	Rate constant for dissociation of NADPH from NADPH Oxidase	11.624	mM	Estimated
75.	l_{nadph}	Rate of formation of intracellular NADPH from other internal sources	8	$mM\ hr^{-1}$	Expected
76.	d_{nadph}	Depletion of NADPH in other intracellular reactions	0.8	hr^{-1}	Expected
77.	V_m^{SOD}	V_{max} of Superoxide Dismutase	$11.4*10^3$	$mM\ hr^{-1}$	[16]
78.	k_{oxrad}^{SOD}	Rate constant for association of free oxygen radical with Superoxide Dismutase	0.054	mM	[16]
79.	V_m^{GTHP}	V_{max} of Glutathione Peroxidase	0.438	$mM\ hr^{-1}$	[17, 18] (ranges: 0.2874 to 2.697 mM/hr)
80.	k_{gsh}^{GTHP}	Rate constant for association of GSH with Glutathione Peroxidase	0.2	mM	[19]
81.	d_{gsh}	Natural decay of intracellular reduced glutathione	0.00016	hr^{-1}	Expected

82.	$k_{h_2o_2}^{GTHP}$	Rate constant for association of H_2O_2 with Glutathione Peroxidase	0.45	mM	[19]
83.	$k_{i(h_2o_2)}^{GTHP}$	Rate constant for dissociation of H_2O_2 from Glutathione Peroxidase	5.3677	mM	Estimated
84.	$d_{h_2o_2}$	Decay of intracellular hydrogen peroxide in other cellular processes	0.001	hr^{-1}	Expected
85.	V_m^{GTHO}	V_{max} of Glutathione Oxidoreductase	0.00216	$mM\ hr^{-1}$	[20]
86.	k_{nadph}^{GTHO}	Rate constant for association of NADPH with Glutathione Oxidoreductase	0.063	mM	[21]
87.	k_{gssg}^{GTHO}	Rate constant for association of GSSG with Glutathione Oxidoreductase	0.154	mM	[21]
88.	$k_{i(gssg)}^{GTHO}$	Rate constant for dissociation of GSSG from Glutathione Oxidoreductase	5.05	mM	Estimated
89.	d_{gssg}	Natural decay of intracellular oxidized glutathione	0.00020	hr^{-1}	Expected
90.	V_m^{XCT}	V_{max} of Cystine-glutamate antiporter	0.001272	$mM\ hr^{-1}$	[22]
91.	$k_{i(cys_e)}^{XCT}$	Rate constant for dissociation of Cystine from Cystine-glutamate antiporter	0.0249	mM	[23]
92.	k_{glut}^{XCT}	Rate constant for association of Glutamate with Cystine-glutamate antiporter	0.084	mM	[24]
93.	$k_{cys_e}^{XCT}$	Rate constant for association of Cystine with Cystine-glutamate antiporter	0.084	mM	[24]
94.	l_{cys_e}	Rate of cystine influx into the ECM via other cellular processes	3	$mM\ hr^{-1}$	Expected
95.	d_{cys_e}	Depletion of extracellular cystine into other cellular processes	0.6	hr^{-1}	Expected
96.	L_{oxy}	Rate of Oxygen diffusing into the ECM and available for cellular uptake	10	$mM\ hr^{-1}$	Expected
97.	d_{oxy}	Utilization of Oxygen by other cell types	0.7	hr^{-1}	Expected
98.	$V_m^{O_2}$	V_{max} of external Oxygen uptake	15.278	$mM\ hr^{-1}$	[25]
99.	$k_m^{O_2}$	K_m of external Oxygen uptake	164	mM	[2]
100.	d_{in}	Utilization of Oxygen in other intracellular reactions	50.313	hr^{-1}	Estimated
101.	$l_{gly(e)}$	Rate of glycine influx into the ECM via other cellular processes	4	$mM\ hr^{-1}$	Expected
102.	$d_{gly(e)}$	Depletion of extracellular glycine into other cellular processes	0.5	hr^{-1}	Expected

103.	V_m^{GLYex}	V_{max} of Glycine Exchanger	0.0287	$mM\ hr^{-1}$	[26]
104.	k_m^{GLYex}	K_m of Glycine Exchanger	0.029	mM	[26, 27]
105.	d_{gly}	Natural decay of intracellular glycine	0.0586	hr^{-1}	Expected
106.	V_m^{G6PDH}	V_{max} of Glucose-6-phosphate dehydrogenase	5.337	$mM\ hr^{-1}$	Estimated
107.	$k_{i(g6p)}^{G6PDH}$	Rate constant for dissociation of G6P from Glucose-6-phosphate dehydrogenase	6.904	mM	Estimated
108.	k_{nadp}^{G6PDH}	Rate constant for association of NADP with Glucose-6-phosphate dehydrogenase	4.9725	mM	Estimated
109.	k_{g6p}^{G6PDH}	Rate constant for association of G6P with Glucose-6-phosphate dehydrogenase	6.627	mM	Estimated
110.	$k_{ii(atp)}^{G6PDH}$	Inhibition constant of ATP for Glucose-6-phosphate dehydrogenase	6.0885	mM	Estimated
111.	l_{nadp}	Rate of formation of intracellular $NADP^+$ from other internal sources	6	$mM\ hr^{-1}$	Expected
112.	d_{nadp}	Depletion of $NADP^+$ in other intracellular reactions	0.5	hr^{-1}	Expected
113.	V_m^{CR}	V_{max} of Cystine Reductase	12	$mM\ hr^{-1}$	Estimated
114.	$k_{i(cys)}^{CR}$	Rate constant for dissociation of Cystine from Cystine Reductase	10	mM	Estimated
115.	k_{nadph}^{CR}	Rate constant for association of NADPH with Cystine Reductase	5.536	mM	Estimated
116.	k_{cys}^{CR}	Rate constant for association of Cystine with Cystine Reductase	5.1175	mM	Estimated
117.	d_{cysl}	Natural decay of intracellular cysteine	0.0517	hr^{-1}	Expected
118.	l_{atp}	Rate of formation of intracellular ATP from other internal reactions	8	$mM\ hr^{-1}$	[28]
119.	d_{atp}	Depletion of ATP in other intracellular reactions	0.5	hr^{-1}	Expected
120.	l_{adp}	Rate of formation of intracellular ADP from other internal reactions	0.8	$mM\ hr^{-1}$	[29]
121.	d_{adp}	Depletion of ADP in other intracellular reactions	0.4	hr^{-1}	Expected
122.	l_{nadh}	Rate of formation of intracellular NADH from other internal sources	5	$mM\ hr^{-1}$	Expected
123.	d_{nadh}	Depletion of NADH in other intracellular reactions	0.7	hr^{-1}	Expected

* Assumptions to the values of rate of influx of glucose and depletion from the extracellular matrix are assumed such that they yielded an extracellular concentration of around 3.9 to 5.4 mM.

** The value has been taken from Brenda and it ranges between 0.37 mM to 0.76 mM [30, 31]
*** The values are assumed based on the reported value of V_m^{DHFR} and V_m^{MTHFR} to be 5000 μM/hr (or 5 mM/hr) [32].
[#] The value ranges around the reported value.

Table S2: Initial values to all the variables considered in the model and their reported range in the biological systems have been listed below.

Variable	Description	Reported Range	Initial Value	Reference
glc_e	Extracellular Glucose	2.0 – 4.0 mM	4 mM	[33]
glc_c	Cytoplasmic Glucose	1.0 – 7.0 mM	4 mM	[34]
$g6p$	Glucose-6-phosphate	~ 0.12 mM	0.38 mM	[35]
$f6p$	Fructose-6-phosphate	~ 0.016 mM	0.016 mM	[36]
$f16bp$	Fructose-1,6-bisphosphate	~ 0.0076 mM	0.0076 mM	[36]
gap	Glyceraldehyde Phosphate	~ 0.0076 mM	0.0076 mM	[36]
$dhap$	Dihydroacetone phosphate	~ 0.14 mM	0.14 mM	[36]
$1,3bpg$	1,3-bisphosphoglycerate	~ 0.0004 mM	0.0004 mM	[36]
$3pg$	3-phosphoglycerate	~ 0.045 mM	0.045 mM	[36]
$3php$	3-Phosphohydroxypyruvate	0.01 to 0.4 mM	0.45 mM	[37]
$pser$	Phosphoserine	~ 0.446 mM	0.446 mM	[38]
ser	Serine	0.5 – 1.0 mM	0.7 mM	[34]
gly	Cytoplasmic Glycine	4.4 – 8.4 mM	5 mM	[34]
thf	Tetrahydrofolate		2.5 mM	Expected
$mlthf$	Methyl-tetrahydrofolate		2.5 mM	Expected
$glut_e$	Extracellular Glutamate	0.00002 - 0.02 mM	0.02 mM	[39]
$glut$	Cytoplasmic Glutamate	2.6 – 4.6 mM	2.5 mM	[34]
cys_e	Extracellular Cystine	~1.6 mM	1.5 mM	[40]
cys	Cytoplasmic Cystine		3.6 mM	Expected
$cysL$	Cysteine	0.5 – 5 mM	1.6 mM	[34]
$glucys$	ɣ-glutamyl cysteine		2.5 mM	Expected
gsh	Reduced Glutathione	2.0 – 3.0 mM	3.83 mM	[41]
$gssg$	Oxidised Glutathione	0.024 – 3.0 mM	0.5 mM	[42]
h_2o_2	Hydrogen peroxide	0.01 – 2 mM	1.55 mM	[43, 44]
$oxrad$	Oxygen free radicals		0.05 mM	Expected
atp	Adenosine tri-phosphate	1.5 – 6.0 mM	1.54 mM	[35]
adp	Adenosine di-phosphate	~ 0.27 mM	2.7 mM	[36]
nad	Nicotinamide adenine Dinucleotide (oxidized)	~ 0.05840 mM	0.0584 mM	[36]
$nadh$	Nicotinamide adenine Dinucleotide (reduced)	~ 0.03060 mM	0.03060 mM	[36]
$nadp^+$	Nicotinamide adenine dinucleotide phosphate	~ 0.0002 mM	0.0002 mM	[36]

	(oxidized)			
nadph	Nicotinamide adenine dinucleotide phosphate (reduced)	~ 0.0658 mM	0.0658 mM	[36]
akg	α-ketoglutarate	~ 0.157 mM	1.4 mM	[38]
$O_{2(ex)}$	Extracellular oxygen	12 - 22mM	15 mM	[25]
O_2	Intracellular oxygen		5 mM	Expected
gly_e	Extracellular glycine	0.2-2 mM	0.2 mM	[26]

5. Parameter estimation:

Parameter estimation of 23 unknown parameters was done using the Delayed Rejection Adaptive Metropolis (DRAM) algorithm of Markov Chain Monte Carlo (MCMC) Toolbox in MATLAB 2017a. The parameter distribution plot of the 23 parameters has been shown in Figure S1A and the trace plot till 5 lakh chains has been shown in Figure S1B. The model was re-evaluated for available data for glutamate exchange in astrocytes [45] to estimate the value of parameters V_m^{GLUTEX} and k_m^{GLUTEX}. The predictive plot for the same has been provided in Figure S2A and the distribution plot and trace plot of estimated parameters have been shown in Figure S2B and S2C respectively.

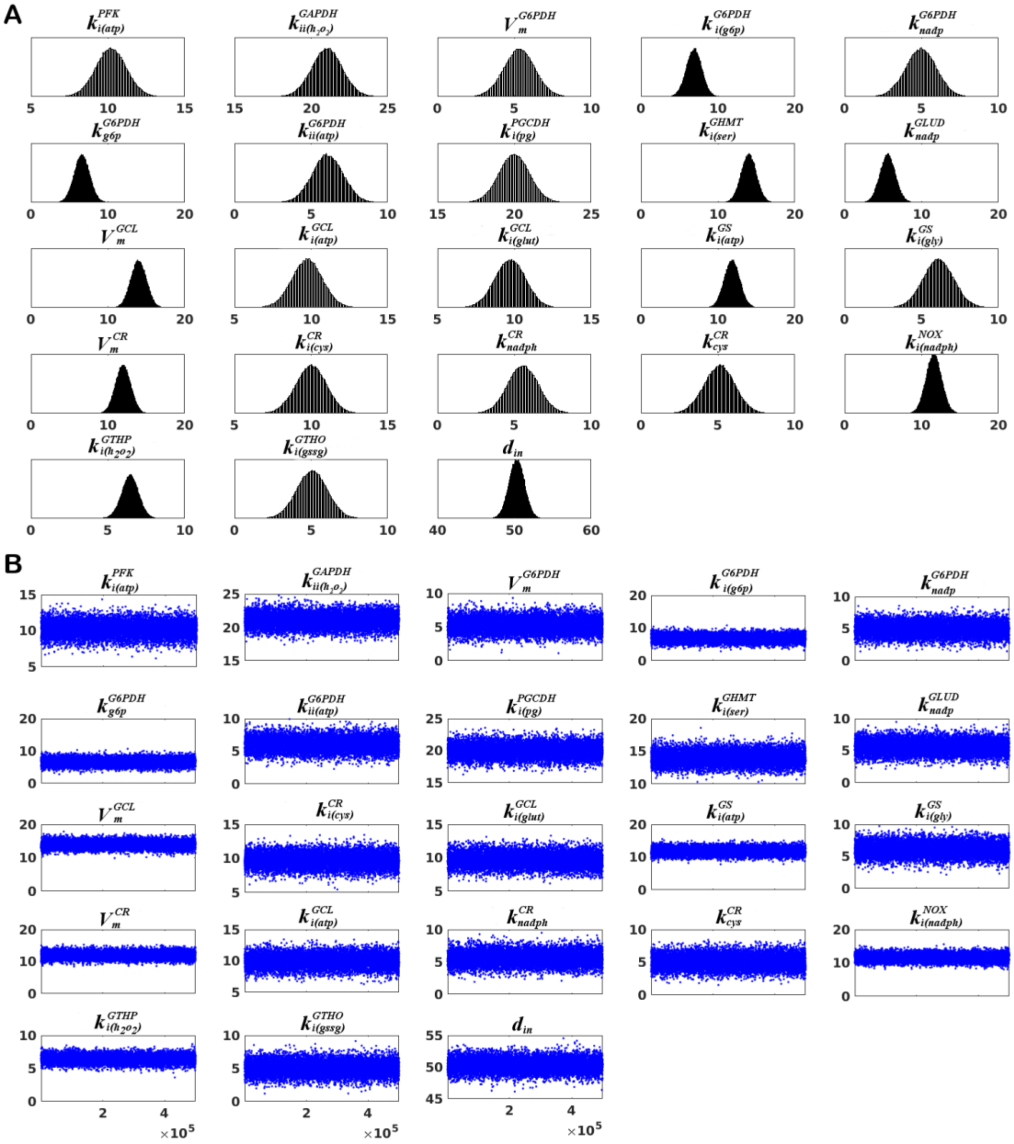

Figure S1: A. Parameter distribution plot of the estimated parameters within the minimum and maximum range provided for the simulations, B. Trace plots of the estimated parameters generated after 5 lakh chains of MCMC DRAM.

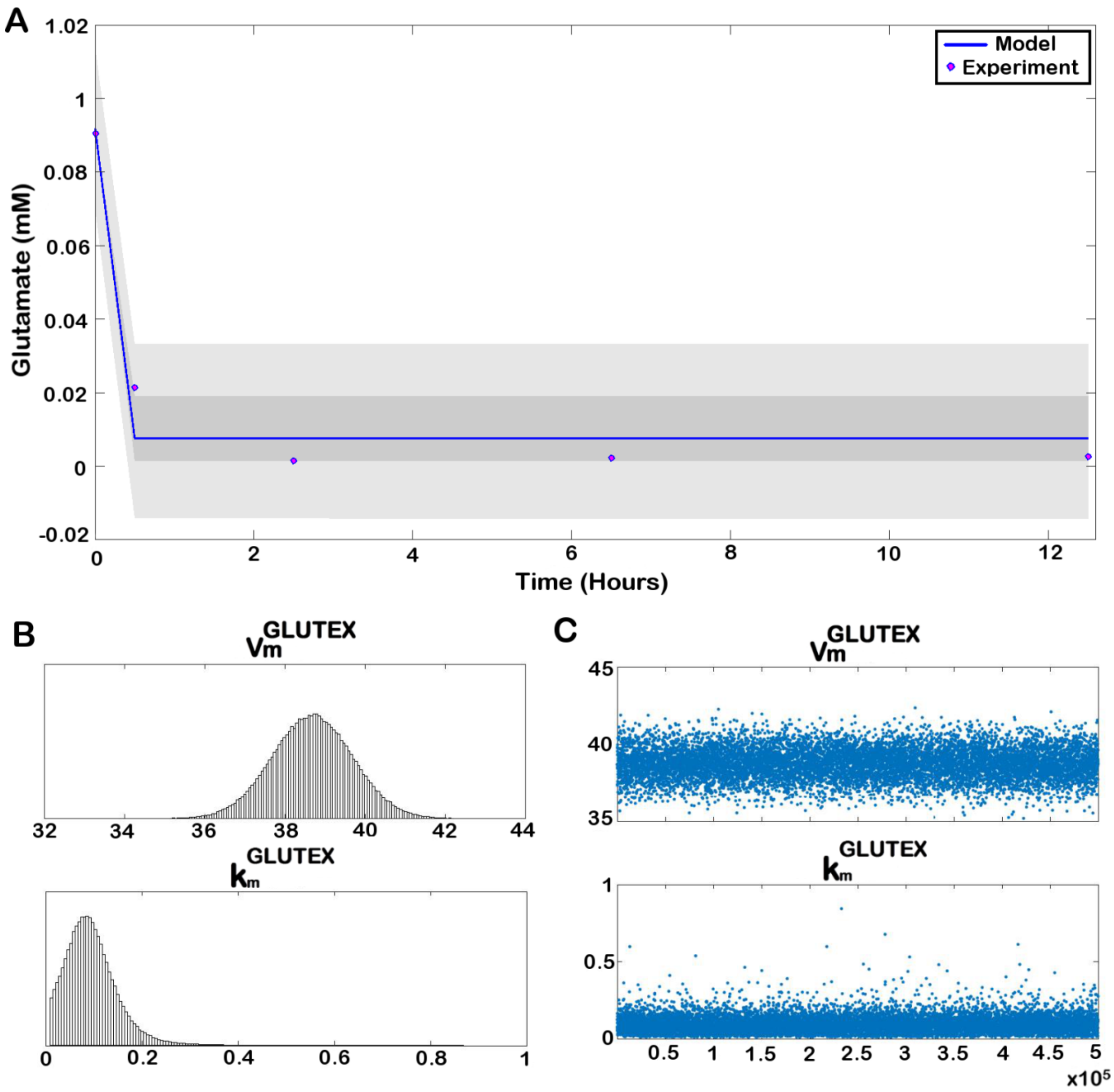

Figure S2: A. Predictive plot demonstrating the fitted model for glutamate exchange and uncertainty regions; B. Parameter distribution curve of the estimated parameters; C. Trace plots of the estimated parameters generated after 5 lakh chains of MCMC DRAM.

6. Sensitivity Analysis:

Extended Fourier Amplified Sensitivity Test (eFAST) algorithm was used for identifying sensitive parameters to the system. The sensitivity analysis was carried out using the whole set of parameters [k=123]. 150 samples were chosen per search curve and the resampling of the search curves was carried out 5 times [NS =150, NR =5]. Hence, the total number of model simulations N=(k+1)*Ns*NR=93000. Sensitivity analyses were performed for normal glial, hypoxic and glioma scenarios created in the model. Supplementary Figure 3 shows the sensitivity index (Si) of the sensitive parameters for the variables gsh (A-C), $gssg$ (D-F), h_2o_2 (G-I) and $oxrad$ (J-L)

under normal glial, hypoxic and glioma scenarios respectively. The sensitivity indices of the parameters were captured for multiple time points (1hr, 2hrs, 4hrs, 8hrs, 16hrs, 20hrs, 24hrs, 32hrs, 50hrs, 100hrs, 150hrs, and 200hrs) and the difference in Si (if any) was taken into account.

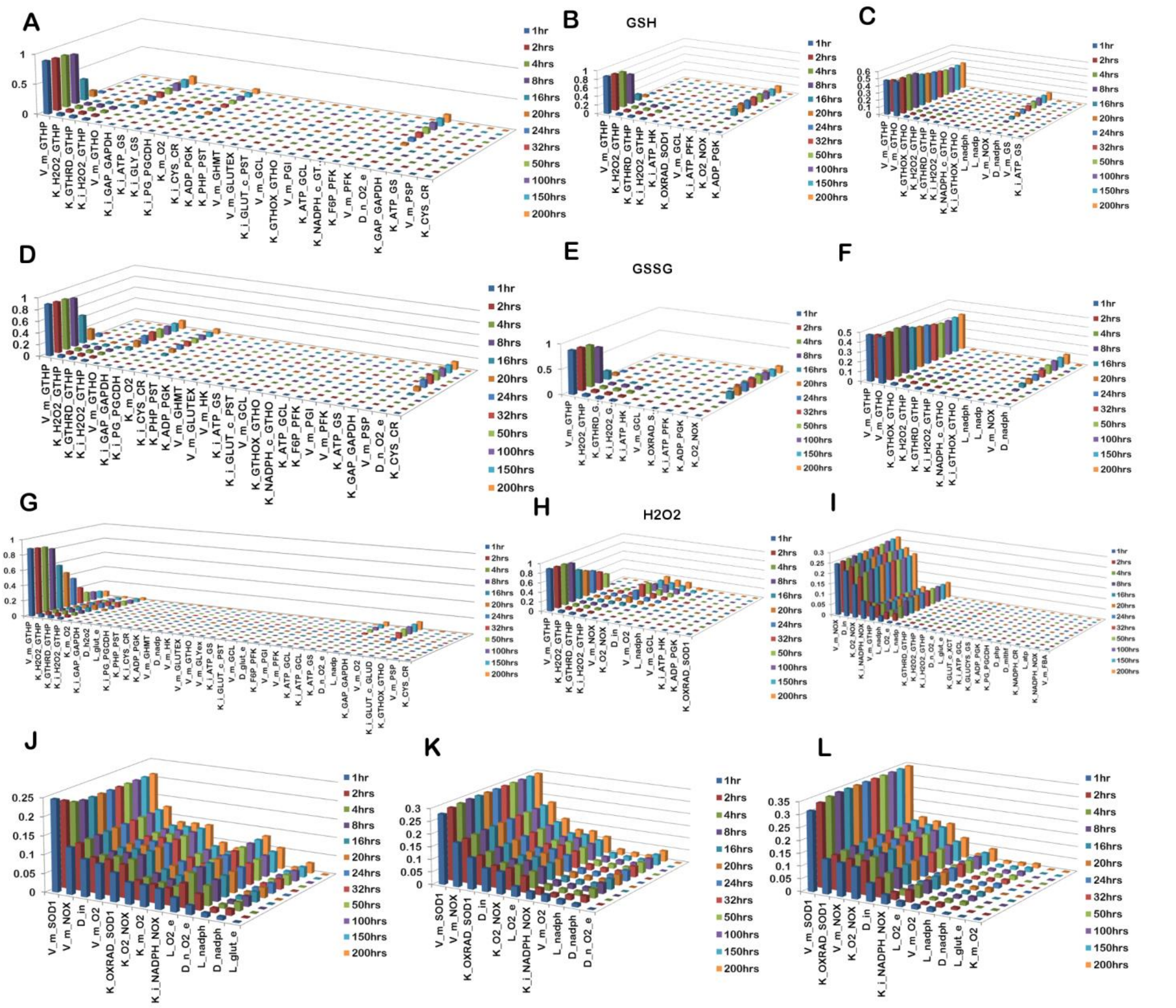

Figure S3: Plots of sensitive parameters for different variables obtained for the three scenarios- normal, hypoxia and hypoxia with multiple mutations respectively: (A-C) *gsh*; (D-F) *gssg*; (G-I) h_2o_2; (J-L) *oxrad*

7. Effect of changing $k_m^{O_2}$ on uptake of Oxygen:

$k_m^{O_2}$ represents the cellular affinity for external oxygen. The model was simulated for different values of $k_m^{O_2}$. A lowering in the value of $k_m^{O_2}$ shows a reduction in the concentration of external oxygen representing an increasing affinity for external oxygen. The changing concentration of external oxygen with changing values of $k_m^{O_2}$ has been shown in Figure S4.

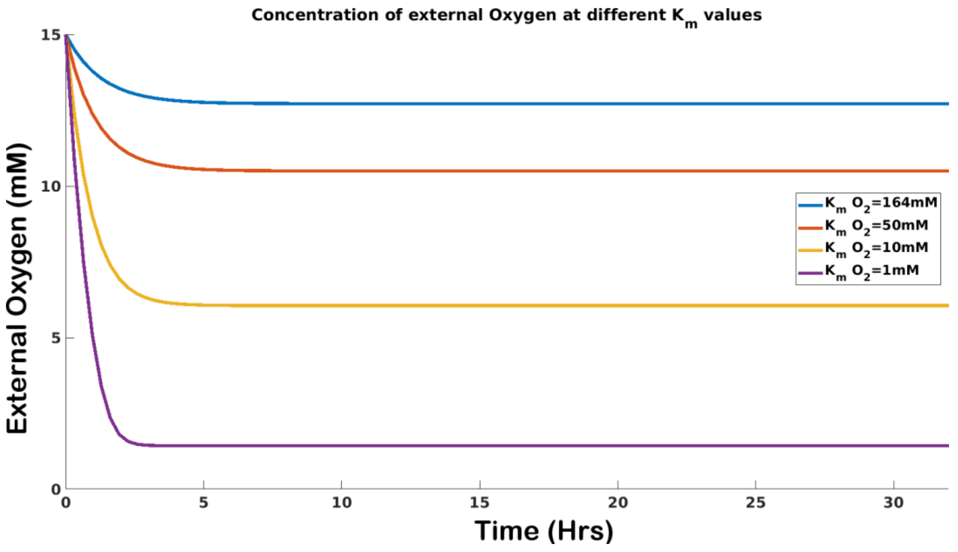

Figure S4: Change in external Oxygen concentration with respect to changing $k_m^{O_2}$.

8. Effect of changing V_m^{GTHO} on $nadph / nadp^+$ ratio:

A change in the $nadph / nadp^+$ ratio was observed depending on the changing value of V_m^{GTHO}. This change is captured in Figure S5. A dip in the $nadph / nadp^+$ ratio is observed for all the values of V_m^{GTHO}. However, with increasing value of V_m^{GTHO}, the decline in the $nadph / nadp^+$ ratio increases which is regained back to normal and the dynamics depend on the value of V_m^{GTHO} and the persisting amount of oxidant (h_2o_2) concentration at any point of time.

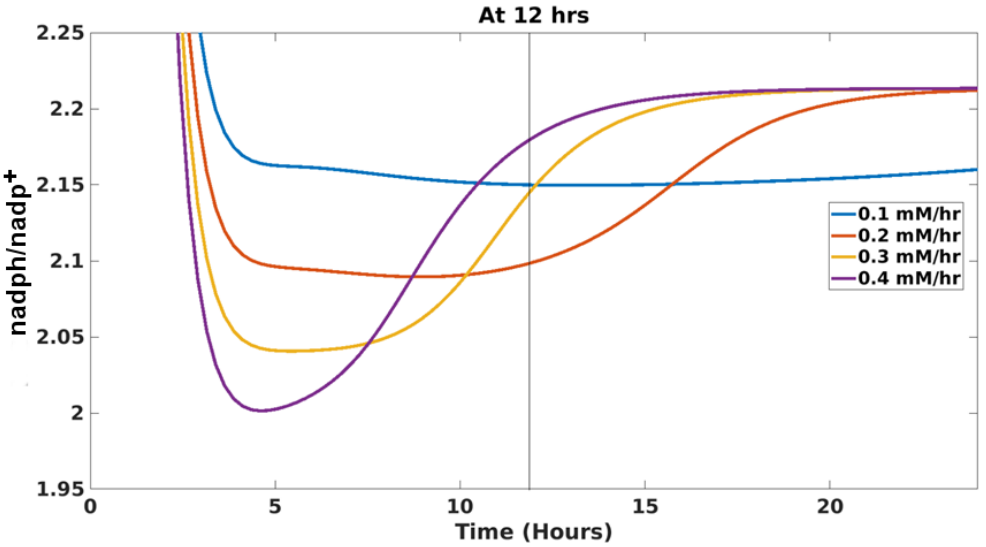

Figure S5: Temporal plot of Varying V_m^{GTHO} and $k_m^{O_2}$ at 1mM over time and its effect on the *nadph/nadp*$^+$ ratio

9. Combinatorial targets for pro- and anti- oxidant therapy:

Glioma scenario was created in the model by incorporating changes in the parameter values V_m^{NOX}, V_m^{GTHO}, V_m^{GTHP} and $k_m^{O_2}$ which were changed to 1*mM* hr^{-1}, 0.2 *mM* hr^{-1}, 0.19 *mM* hr^{-1} and 1 *mM* from 0.0468 *mM* hr^{-1}, 0.5 *mM* hr^{-1}, 0.00216 *mM* hr^{-1} and 164*mM* respectively. Combinatorial variations of sensitive parameters reported in the glioma scenario G11 specified in Table 1 of the main text were made. The values of the parameters were varied between a wide range (0.0001 to 100 units) and changes were observed. Here we have reported the temporal variation in the h_2o_2, *gsh/gssg* and $nadph/nadp^+$ profiles for a particular value of the parameters. The values of the parameters have been specified in Table S2 and the temporal plots have been shown in Figure S6.

Table S3: Values of parameters for which pro- and anti-oxidant effects are obtained in the glioma scenario.

Sr. No.	Parameter 1	Parameter 2	Value of Parameter 1	Value of Parameter 2	Effect
1.	$k_{i(nadph)}^{NOX}$	k_{nadph}^{CR}	1 *mM*	15 *mM*	Pro-oxidant
2.	$k_{i(nadph)}^{NOX}$	k_{nadph}^{NOX}	20 *mM*	10 *mM*	Anti-oxidant
			1 *mM*	0.0001 *mM*	Pro-oxidant
3.	L_{oxy}	k_{nadph}^{CR}	20 *mM* hr^{-1}	15 *mM*	Pro-oxidant
4.	$k_{i(nadph)}^{NOX}$	l_{atp}	0.5 *mM*	20 *mM* hr^{-1}	Pro-oxidant
5.	V_m^{FBA}	L_{oxy}	0.0001 *mM* hr^{-1}	5 *mM* hr^{-1}	Anti-oxidant
6.	k_{glucys}^{GS}	k_{nadph}^{CR}	0.0001 *mM*	0.5 *mM*	Pro-oxidant

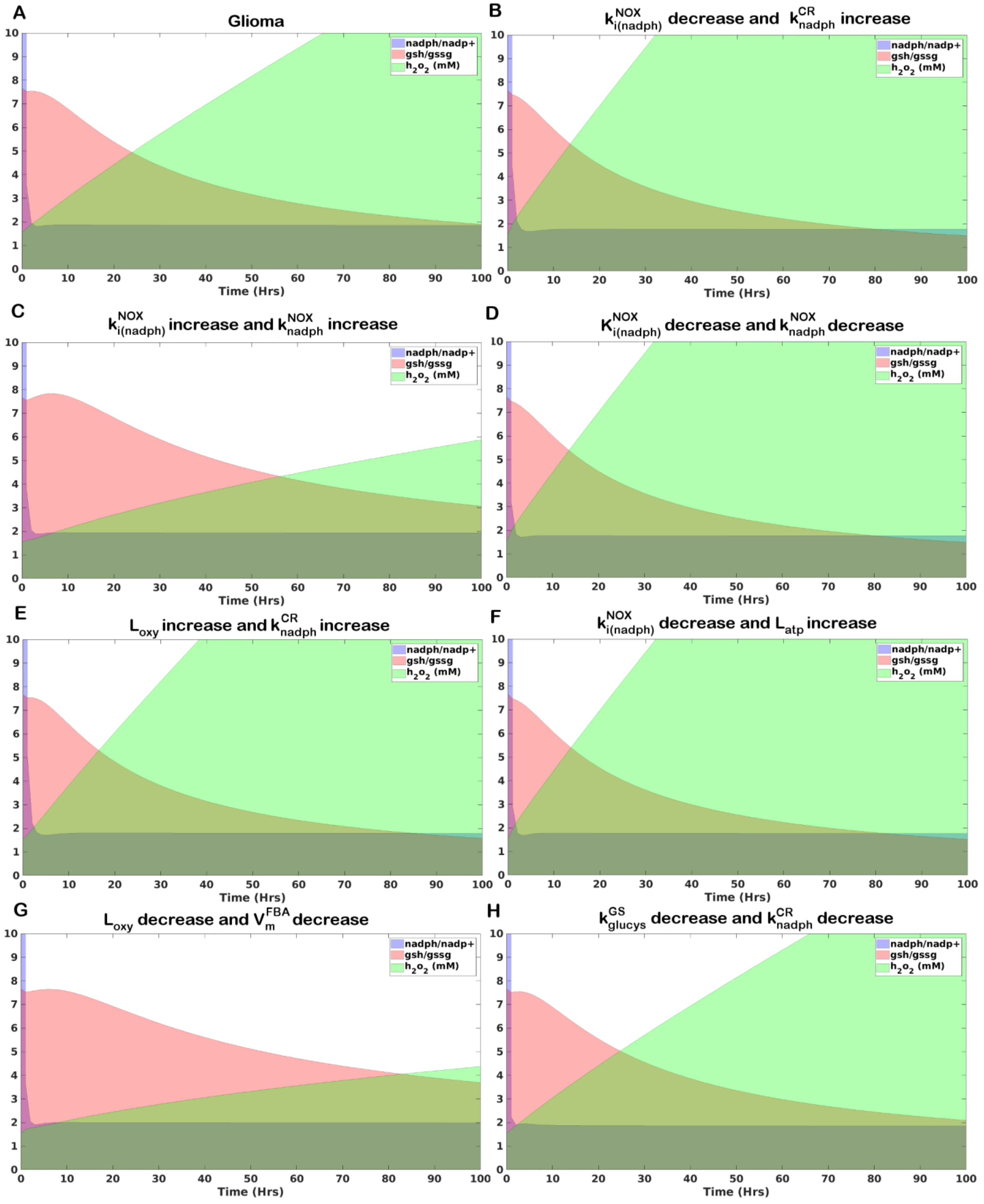

Figure S6: Temporal area plots of changing *nadph/nadp*$^+$ and *gsh/gssg* ratios along with the change in h_2o_2 concentration with a combinatorial variation of parameters made in glioma scenario.

10. References

1. Mulukutla, B.C., Yongky, A., Grimm, S., Daoutidis, P. and Hu, W. S., *Multiplicity of steady states in glycolysis and shift of metabolic state in cultured mammalian cells.* PloS One, 2015. **10**(3): p. e0121561.
2. Shestov, A.A., Shestov, A.A., Liu, X., Ser, Z., Cluntun, A.A., Hung, Y.P., Huang, L., Kim, D., Le, A., Yellen, G., Albeck, J.G. and Locasale, J.W., *Quantitative determinants of aerobic glycolysis identify flux through the enzyme GAPDH as a limiting step*. Elife, 2014. **3**:p. e03342
3. Bell, R.A., Smith, J.C. and Storey, K.B., *Purification and properties of glyceraldehyde-3-phosphate dehydrogenase from the skeletal muscle of the hibernating ground squirrel, Ictidomys tridecemlineatus*. PeerJ, 2014. **2**: p. e634.
4. Tabatabaie, L., De Koning, T.J., Geboers, A.J.J.M., van den Berg, I.E.T., Berger, R. and Klomp, L.W.J., *Novel mutations in 3-phosphoglycerate dehydrogenase (PHGDH) are distributed throughout the protein and result in altered enzyme kinetics*. Hum Mutat, 2009. **30**(5): p. 749-756.
5. Fan, J., Teng, X., Liu, L., Mattaini, K.R., Looper, R.E., Vander Heiden, M.G. and Rabinowitz, J.D., *Human phosphoglycerate dehydrogenase produces the oncometabolite D-2-hydroxyglutarate*. ACS Chem Biol, 2014. **10**(2): p. 510-516.
6. Hart, C.E., Race, V., Achouri, Y., Wiame, E., Sharrard, M., Olpin, S.E., Watkinson, J., Bonham, J.R., Jaeken, J., Matthijs, G. and Van Schaftingen, E., *Phosphoserine aminotransferase deficiency: a novel disorder of the serine biosynthesis pathway.* Am J Hum Genet, 2007. **80**(5): p. 931-937.
7. Collet, J.-F., Stroobant, V. and Van Schaftingen, E., *Mechanistic studies of phosphoserine phosphatase, an enzyme related to P-type ATPases*. J Biol Chem, 1999. **274**(48): p. 33985-33990.
8. Shetty, K.T., *Phosphoserine phosphatase of human brain: partial purification, characterization, regional distribution, and effect of certain modulators including psychoactive drugs*. Neurochem Res, 1990. **15**(12): p. 1203-1210.
9. Vazquez, A., Markert, E.K. and Oltvai, Z.N., *Serine biosynthesis with one carbon catabolism and the glycine cleavage system represents a novel pathway for ATP generation*. PloS One, 2011. **6**(11): p. e25881.
10. Nijhout, H.F., Reed, M.C., Budu, P. and Ulrich, C.M., *A mathematical model of the folate cycle new insights into folate homeostasis*. J Biol Chem, 2004. **279**(53): p. 55008-55016.
11. Willis, M.N., Liu, Y., Biterova, E.I., Simpson, M.A., Kim, H., Lee, J. and Barycki, J.J., *Enzymatic defects underlying hereditary glutamate cysteine ligase deficiency are mitigated by association of the catalytic and regulatory subunits.* Biochemistry -US, 2011. **50**(29): p. 6508-6517.
12. York, M., Kuchel, P.W., Chapman, B.E. and Jones, A., *Incorporation of labelled glycine into reduced glutathione of intact human erythrocytes by enzyme-catalysed exchange. A nuclear-magnetic-resonance study*. Biochem J, 1982. **207**(1): p. 65-72.
13. Njålsson, R., Carlsson, K., Birgit, O.L.I.N., Carlsson, B., Whitbread, L., Polekhina, G., Parker, M.W., Norgren, S., Mannervik, B., Philip, G. and Larsson, A., *Kinetic properties of missense mutations in patients with glutathione synthetase deficiency*. Biochem J, 2000. **349**(Pt 1): p. 275.

14. Gatto Jr, G.J., Ao, Z., Kearse, M.G., Zhou, M., Morales, C.R., Daniels, E., Bradley, B.T., Goserud, M.T., Goodman, K.B., Douglas, S.A. and Harpel, M.R., *NADPH oxidase-dependent and-independent mechanisms of reported inhibitors of reactive oxygen generation*. J Enzym Inhib Med Chem, 2013. **28**(1): p. 95-104.
15. Brandes, R.P., Weissmann, N. and Schröder, K., *Nox family NADPH oxidases: molecular mechanisms of activation*. Free Radical Biology and Medicine, 2014. **76**: p. 208-226.
16. Searcy, D.G., *HS−: O2oxidoreductase activity of Cu, Zn superoxide dismutase.* Arch Biochem Biophys, 1996. **334**(1): p. 50-58.
17. Suravajjala, S., Cohenford, M., Frost, L.R., Pampati, P.K. and Dain, J.A., *Glycation of human erythrocyte glutathione peroxidase: effect on the physical and kinetic properties*. Clin Chim Acta, 2013. **421**: p. 170-176.
18. Lankin, V.Z., Konovalova, G.G., Tikhaze, A.K., Shumaev, K.B., Belova, E.M., Grechnikova, M.A. and Viigimaa, M., *Aldehyde inhibition of antioxidant enzymes in the blood of diabetic patients*. J Diabetes, 2016. **8**(3): p. 398-404.
19. Liu, L., Mao, S.Z., Liu, X.M., Huang, X., Xu, J.Y., Liu, J.Q., Luo, G.M. and Shen, J.C., *Functional mimicry of the active site of glutathione peroxidase by glutathione imprinted selenium-containing protein*. Biomacromolecules, 2007. **9**(1): p. 363-368.
20. Storey, B.T., Alvarez, J.G. and Thompson, K.A., *Human sperm glutathione reductase activity in situ reveals limitation in the glutathione antioxidant defense system due to supply of NADPH*. Mol Reprod Dev, 1998. **49**(4): p. 400-407.
21. Ulusu, N.N. and Tandoğan, B., *Purification and kinetic properties of glutathione reductase from bovine liver*. Mol Cell Biochem, 2007. **303**(1-2): p. 45-51.
22. Bender, A., Reichelt, W., and Norenberg, M., *Characterization of cystine uptake in cultured astrocytes*. Neurochem Int, 2000. **37**(2-3): p. 269-276.
23. Hosoya, K.-I., Tomi, M., Ohtsuki, S., Takanaga, H., Saeki, S., Kanai, Y., Endou, H., Naito, M., Tsuruo, T. and Terasaki, T., *Enhancement of L-cystine transport activity and its relation to xCT gene induction at the blood-brain barrier by diethyl maleate treatment*. J Pharm Exp Ther, 2002. **302**(1): p. 225-231.
24. Thomas, A.G., Sattler, R., Tendyke, K., Loiacono, K.A., Hansen, H., Sahni, V., Hashizume, Y., Rojas, C. and Slusher, B.S., *High-throughput assay development for cystine-glutamate antiporter (xc-) highlights faster cystine uptake than glutamate release in glioma cells*. PloS One, 2015. **10**(8): p. e0127785.
25. Shipley, R., Davidson, A.J., Chan, K., Chaudhuri, J.B., Waters, S.L. and Ellis, M.J., *A strategy to determine operating parameters in tissue engineering hollow fiber bioreactors*. Biotech Bioeng, 2011. **108**(6): p. 1450-1461.
26. Ellory, J., Jones, S. and Young, J., *Glycine transport in human erythrocytes*. J Physiol, 1981. **320**(1): p. 403-422.
27. Aroeira, R.I., Sebastião, A.M. and Valente, C.A., *BDNF, via truncated TrkB receptor, modulates GlyT1 and GlyT2 in astrocytes*. Glia, 2015. **63**(12): p. 2181-2197.
28. Zimmerman, J.J., von Saint André-von Arnim, A. and McLaughlin, J., *Cellular Respiration*, in *Pediatric Critical Care*. 2011, Elsevier. p. 1058-1072.
29. Desmoulin, F., Cozzone, P.J. and Canioni, P., *Phosphorus-31 nuclear-magnetic-resonance study of phosphorylated metabolites compartmentation, intracellular pH and phosphorylation state during normoxia, hypoxia and ethanol perfusion, in the perfused rat liver*. Eur J Biochem, 1987. **162**(1): p. 151-159.

30. Ahn, K.-J., Kim, J.S., Yun, M.J., Park, J.H. and Lee, J.D., *Enzymatic properties of the N- and C-terminal halves of human hexokinase II.* BMB Rep, 2009. **42**(6): p. 350-355.
31. Antoine, M., Boutin, J.A. and Ferry, G., *Binding kinetics of glucose and allosteric activators to human glucokinase reveal multiple conformational states.* Biochemistry, 2009. **48**(23): p. 5466-5482.
32. Reed, M.C., Nijhout, H.F., Neuhouser, M.L., Gregory III, J.F., Shane, B., James, S.J., Boynton, A. and Ulrich, C.M., *A mathematical model gives insights into nutritional and genetic aspects of folate-mediated one-carbon metabolism.* J Nutr, 2006. **136**(10): p. 2653-2661.
33. Silver, I.A. and Erecinska, M., *Extracellular glucose concentration in mammalian brain: continuous monitoring of changes during increased neuronal activity and upon limitation in oxygen supply in normo-, hypo-, and hyperglycemic animals.* J Neurosci, 1994. **14**(8): p. 5068-5076.
34. Behjousiar, A., Kontoravdi, C. and Polizzi, K.M., *In situ monitoring of intracellular glucose and glutamine in CHO cell culture.* PLoS One, 2012. **7**(4): p. e34512.
35. Erwin, I.A. and O'Connell, E., *The Role of Glucose 6-Phosphate in the Regulation Glucose Metabolism in Human Erythrocytes.* J Biol Chem, 1964. **239**: p. 12-17.
36. Jamshidi, N. and Palsson, B.Ø., *Mass action stoichiometric simulation models: incorporating kinetics and regulation into stoichiometric models.* Biophys J, 2010. **98**(2): p. 175-185.
37. Klomp, L.W., de Koning, T.J., Malingré, H.E., van Beurden, E.A., Brink, M., Opdam, F.L., Duran, M., Jaeken, J., Pineda, M., van Maldergem, L. and Poll-The, B.T., *Molecular characterization of 3-phosphoglycerate dehydrogenase deficiency—a neurometabolic disorder associated with reduced L-serine biosynthesis.* Am J Hum Genet, 2000. **67**(6): p. 1389-1399.
38. Snell, K. and Fell, D.A., *Metabolic control analysis of mammalian serine metabolism.* Adv Enzyme Regul, 1990. **30**: p. 13-32.
39. Kalivas, P., *Extracellular glutamate: functional compartments operate in different concentration ranges.* Front Syst Neurosci, 2011. **5**: p. e94.
40. Bannai, S., *Transport of cystine and cysteine in mammalian cells.* Biochimica et Biophysica Acta (BBA)-Rev Biomembranes, 1984. **779**(3): p. 289-306.
41. Aoyama, K. and Nakaki, T., *Impaired glutathione synthesis in neurodegeneration.* Int J Mol Sci, 2013. **14**(10): p. 21021-21044.
42. Zitka, O., Skalickova, S., Gumulec, J., Masarik, M., Adam, V., Hubalek, J., Trnkova, L., Kruseova, J., Eckschlager, T. and Kizek, R., *Redox status expressed as GSH: GSSG ratio as a marker for oxidative stress in paediatric tumour patients.* Oncol Lett, 2012. **4**(6): p. 1247-1253.
43. Nakamura, J., Purvis, E.R. and Swenberg, J.A., *Micromolar concentrations of hydrogen peroxide induce oxidative DNA lesions more efficiently than millimolar concentrations in mammalian cells.* Nucleic Acids Res, 2003. **31**(6): p. 1790-1795.
44. Clément, M.-V. and Pervaiz, S., *Intracellular superoxide and hydrogen peroxide concentrations: a critical balance that determines survival or death.* Redox Rep, 2001. **6**(4): p. 211-214.
45. Ye, Z.-C. and Sontheimer, H., *Glioma cells release excitotoxic concentrations of glutamate.* Cancer Res, 1999. **59**(17): p. 4383-4391.
