# Supplementary material for: Differential suitability of reactive oxygen species and the role of glutathione in regulating paradoxical behavior in gliomas: A mathematical perspective
Source: PLoS One. 2020 Jun 25;15(6):e0235204. doi: 10.1371/journal.pone.0235204 (PMC7316271; doi:10.1371/journal.pone.0235204)
Supplement: Attachment — Submitted filename: ResponsetoReviewers.docx [file pone.0235204.s002.docx]

**Answer to Reviewers’ comments**

**To**

**The Editor**

**PLoS ONE**

First, we would like to sincerely thank you for giving us the opportunity to revise our manuscript. The manuscript is now thoroughly revised following the suggestions/comments made by the Reviewers (**Track Changes in the Revised Manuscript**) and the point-by-point answers are given below.

We hope this revised version will satisfy you and the reviewers and will be acceptable to the journal.

**--------------------------------------------------------------------------------------------------------------------**

**Reviewer comments:**

**Editorial Staffs:**

*Our editorial staff has assessed your submission, and we have significant concerns about the grammar, usage, and overall readability of the manuscript. We believe that the manuscript is much more likely to be accepted if it is easy for the reviewers to read and understand. We therefore request that you revise the text to fix the grammatical errors and improve the overall readability of the text before we send it for review.*

*We suggest you have a fluent, preferably native, English-language speaker thoroughly copyedit your manuscript for language usage, spelling, and grammar. If you do not know anyone who can do this, you may wish to consider employing a professional scientific editing service.*

**Answer:** We sincerely thank the Editorial Staffs for the prompt review of the manuscript and for the valuable comments. We have performed a thorough scrutiny of the manuscript to improvise on the points raised by both the reviewers. We have thoroughly checked the entire manuscript and the supplementary text file to reduce the grammatical and typographical errors of the manuscript, as has been rightly pointed out. Also we have improved the overall readability of the manuscript by changing few sentences and grammatical corrections (please check the Track-changed version of the manuscript).

Kindly note that, apart from manual checking by a native, English-language speaker, we have also used the Grammarly software (<https://www.grammarly.com/edu>; License and access provided to our Institute Library, CSIR-National Chemical Laboratory through NKRC consortia) and the changes are incorporated in the revised version of the manuscript.

We hope that this revised version will satisfy the reviewer and will be acceptable for publication in PLoS ONE.
