# Supplementary material for: Differential suitability of reactive oxygen species and the role of glutathione in regulating paradoxical behavior in gliomas: A mathematical perspective
Source: PLoS One. 2020 Jun 25;15(6):e0235204. doi: 10.1371/journal.pone.0235204 (PMC7316271; doi:10.1371/journal.pone.0235204)
Supplement: Attachment — Submitted filename: Response to Reviewers.docx [file pone.0235204.s003.docx]

**Response to Reviewer**

**To,**

**Prof. Salvatore V Pizzo**

**Academic Editor**

**PLOS ONE**

Dear Prof. Pizzo,

First we would like to sincerely thank you for giving us the opportunity to perform the revision of our manuscript. We also thank the Reviewer for a detailed and critical review of our manuscript highlighting its deficiencies along with indicating the usefulness of our endeavour. The comments are insightful and have brought out several points that were not clearly stated in the earlier version of the paper. We are also thankful to the Reviewer for recognizing the importance of our work and effort, and advising us to submit the revised version of the manuscript. The manuscript is now thoroughly revised following the suggestions/comments made by the Reviewer (**Highlighted in the revised Manuscript, Marked-up Copy**) and the point to point answers are given below.

We hope this revised version will satisfy you and the reviewer and will be acceptable to the journal.

**----------------------------------------------------------------------------------------------------**

REPLY TO REVIEWERS’ COMMENTS

***Reviewer #1:*** *The manuscript by Bhowmick and Sarkar describes an ODE model of the coordinated function of several metabolic pathways involved in the regulation of the redox balance of the cell. The aim of the model is to study various ROS manipulation strategies in cancerous cells (compared to normal conditions), and, eventually, to evaluate the applicability and effectiveness of ROS promotion or ROS depletion strategies in cancer therapeutics. Special emphasis is laid on glial and glioma cells.
The manuscript is well structured and provides a thorough description of the model and the corresponding analyses. In particular the comprehensive appendix provides valuable information and completes the model specification. All in all, the work appears sound and valid.
Therefore I only have a few minor comments.*

**Reply:** We thank the Reviewer for appreciating our efforts.

***Comment 1:*** *The validation of a mathematical or computational model is a very critical step and should be performed very carefully and described in detail. In its current form this aspect comes a bit short. The authors should provide a separate paragraph and a more thorough description of their efforts in validating the entire model and/or, if done, distinct components of it. This should be complemented by a short discussion including statements under which conditions and for which cell types the authors consider their model as valid and predictive.*

**Reply:** Following the Reviewer's suggestion, a separate paragraph describing the validation of the model has been included in the manuscript. The earlier subsection ‘**2.4. Parameter estimation and validation with experimental data’** has been split and a new subsection **‘2.5.** **Model Validation with experimental data’** under the **Materials and Methods** section has been added. A detailed description of model validation with proper references has been included in this subsection. Also, a short discussion on the conditions and cell types for which the model remains valid and predictive has been added by the end of this subsection. (Revised Manuscript, Line #243-295)

***Comment 2:*** *In line with the previous aspect, the model has only been fitted against glial cells and glioma conditions - how do these results apply to other cancer types? Are the mechanisms described in the manuscript applicable to any other tumor type?*

**Reply**: As hypoxia and oxidative stress are characteristics of most tumors and antioxidant machinery remains central in maintaining redox balances in all cell types, the present model can be used to simulate various scenarios related to redox imbalances in other types of cancers as well. A note on the applicability of the model in other cancers has been included in the **Discussion** section (Manuscript, Line #761-769).

***Comment 3:*** *The introduction is informative, but also a bit lengthy, and could be straightened at some points.*

**Reply**: As per the Reviewer’s suggestion, few of the details in the **Introduction** have been shortened and made straight. The changes can be tracked in the “**Introduction**” section of the ‘**Revised Manuscript with Track Changes’** file.

***Comment 4:*** *The current way the parameter variation experiments of GTHP is described can cause some confusion: in table S1 the physiological range of the parameter Vm_[GTHP] is given as "(ranges: 0.2874 to 2.697 mM/hr)", which is in strong contrast to the parameter variation given in the manuscript (from 0.001mM/hr to 1.5 mM/hr). Here the authors should clearly communicate that (very) low expression of GTHP is characteristic for glioma cells, in particular because interesting changes in model behaviour are clearly below the normal conditions and occur up to 0.3mM/hr.*

**Reply**: We thank the Reviewer for this useful comment. Through our simulation, we could indeed observe that a diminished expression of *GTHP* is a characteristic of gliomas. This has also been observed in other literatures, although the interpretations were made for brain tumors in general. This interpretation was missing in the earlier version of the manuscript and has been included now under the **Results** section (Manuscript, Line #396-404).

***Comment 5:*** *Why has GTHO only been varied in combination with other parameters?*

**Response**: For the analysis of cellular behavior under glioma scenario, we changed the parameter values of $V_{m}^{NOX}$, $V_{m}^{GTHO}$, $V_{m}^{GTHP}$and $k_{m}^{O_{2}}$ as the system remained robust for most of the other parameter changes. We started with varying each of the parameters in combination with the other parameters, but could observe significant differences in *h_2_o_2_* concentration only when glutathione reductase (*GTHO)* was varied in combination with other parameters. To understand this model property, we searched literature to check if varying expression of *GTHO* is an observable trait of gliomas. Zhu *et. al.* (J Neurochem. 2018; 144(1):93-10), in their study, they have shown that different GBM cell lines have different level of *GTHO* expression and overexpression of *GTHO* mediates drug resistance in these cells. Whereas, *GTHO* knockdown resensitize *GTHO* overexpressed cells to drug treatment. We corroborated this understanding with our model simulations to observe that varying *GTHO* significantly affect the thiol ratio of the gliomas along with significant difference in *h_2_o_2_* concentration. This can be attributed to the biochemical property of the enzyme glutathione reductase which is to readily metabolize *gssg* to *gsh* through *nadph-nadp^+^* mediated reaction so that *gsh* pool remains available for neutralizing oxidants maintaining a high *gsh/gssg* ratio. Thus, we propose that, at high expression of *GTHO*, the increased level of *gsh/gssg* ratio helps the cell to evade programmed cell death which would otherwise lead to apoptosis by the induction of toxicity due to uncontrolled increase in *h_2_o_2_* levels. However, with lower expression of *GTHO*, a sharp decline in the *gsh/gssg* ratio drives the cell towards an apoptotic fate. A note on this has been added in the manuscript (Revised Manuscript, Line #560-567).

***Comment 6:*** *Finally, it would be nice to have an endogenous mechanism causing the higher oxygen demand, instead of regulating the cellular oxygen demand by its affinity. This is fine for the current state of the model, but for future work such extension would be an interesting aspect.*

**Reply**: We again thank the Reviewer for this suggestion. Endogenous factors like HIF1α expression, faulty Oxidative Phosphorylation, etc., in addition to the current state of the model will be considered in the future extension of this model. A note on this has been included in the Conclusion section (Revised Manuscript, Line #783-786).

We hope that the above replies will satisfy the reviewer and the manuscript will be acceptable for publication.
