# Supplementary material for: Differential suitability of reactive oxygen species and the role of glutathione in regulating paradoxical behavior in gliomas: A mathematical perspective
Source: PLoS One. 2020 Jun 25;15(6):e0235204. doi: 10.1371/journal.pone.0235204 (PMC7316271; doi:10.1371/journal.pone.0235204)
Supplement: Peer review history

# Author comment: Author response to previous submission

**DOI:** 10.1371/journal.pone.0235204.r001

11 Oct 2019

Attachment Submitted filename: ResponsetoReviewers.docx Click here for additional data file.

# Peer review: Decision Letter 0

**DOI:** 10.1371/journal.pone.0235204.r002

**Authors:** Salvatore V Pizzo

9 Apr 2020

PONE-D-19-27485

Differential suitability of Reactive Oxygen Species and the Role of Glutathione in regulating paradoxical behavior in Gliomas: A mathematical perspective

PLOS ONE

Dear Dr. Sarkar,

Thank you for submitting your manuscript to PLOS ONE. After careful consideration, we feel that it has merit but does not fully meet PLOS ONE’s publication criteria as it currently stands. Therefore, we invite you to submit a revised version of the manuscript that addresses the points raised during the review process. process.

As you can see, the Reviewer had minor issues with the manuscript that must be addressed before we can consider a revised manuscript.

We would appreciate receiving your revised manuscript by May 24 2020 11:59PM. When you are ready to submit your revision, log on to https://www.editorialmanager.com/pone/ and select the 'Submissions Needing Revision' folder to locate your manuscript file.

If you would like to make changes to your financial disclosure, please include your updated statement in your cover letter.

To enhance the reproducibility of your results, we recommend that if applicable you deposit your laboratory protocols in protocols.io, where a protocol can be assigned its own identifier (DOI) such that it can be cited independently in the future. For instructions see: http://journals.plos.org/plosone/s/submission-guidelines#loc-laboratory-protocols

Please include the following items when submitting your revised manuscript:

- A rebuttal letter that responds to each point raised by the academic editor and reviewer(s). This letter should be uploaded as separate file and labeled 'Response to Reviewers'.
- A marked-up copy of your manuscript that highlights changes made to the original version. This file should be uploaded as separate file and labeled 'Revised Manuscript with Track Changes'.
- An unmarked version of your revised paper without tracked changes. This file should be uploaded as separate file and labeled 'Manuscript'.

Please note while forming your response, if your article is accepted, you may have the opportunity to make the peer review history publicly available. The record will include editor decision letters (with reviews) and your responses to reviewer comments. If eligible, we will contact you to opt in or out.

We look forward to receiving your revised manuscript.

Kind regards,

Salvatore V Pizzo

Academic Editor

PLOS ONE

Journal Requirements:

When submitting your revision, we need you to address these additional requirements.

1. Please ensure that your manuscript meets PLOS ONE's style requirements, including those for file naming. The PLOS ONE style templates can be found at

http://www.journals.plos.org/plosone/s/file?id=wjVg/PLOSOne_formatting_sample_main_body.pdf and http://www.journals.plos.org/plosone/s/file?id=ba62/PLOSOne_formatting_sample_title_authors_affiliations.pdf

[Note: HTML markup is below. Please do not edit.]

Reviewers' comments:

Reviewer's Responses to Questions

**Comments to the Author**

1. Is the manuscript technically sound, and do the data support the conclusions?

The manuscript must describe a technically sound piece of scientific research with data that supports the conclusions. Experiments must have been conducted rigorously, with appropriate controls, replication, and sample sizes. The conclusions must be drawn appropriately based on the data presented. 

Reviewer #1: Yes

**********

2. Has the statistical analysis been performed appropriately and rigorously? 

Reviewer #1: No

**********

3. Have the authors made all data underlying the findings in their manuscript fully available?

The PLOS Data policy requires authors to make all data underlying the findings described in their manuscript fully available without restriction, with rare exception (please refer to the Data Availability Statement in the manuscript PDF file). The data should be provided as part of the manuscript or its supporting information, or deposited to a public repository. For example, in addition to summary statistics, the data points behind means, medians and variance measures should be available. If there are restrictions on publicly sharing data—e.g. participant privacy or use of data from a third party—those must be specified.

Reviewer #1: Yes

**********

4. Is the manuscript presented in an intelligible fashion and written in standard English?

PLOS ONE does not copyedit accepted manuscripts, so the language in submitted articles must be clear, correct, and unambiguous. Any typographical or grammatical errors should be corrected at revision, so please note any specific errors here.

Reviewer #1: Yes

**********

5. Review Comments to the Author

Please use the space provided to explain your answers to the questions above. You may also include additional comments for the author, including concerns about dual publication, research ethics, or publication ethics. (Please upload your review as an attachment if it exceeds 20,000 characters)

Reviewer #1: The manuscript by Bhowmick and Sarkar describes an ODE model of the coordinated function of several metabolic pathways involved in the regulation of the redox balance of the cell.

The aim of the model is to study various ROS manipulation strategies in cancerous cells (compared to normal conditions), and, eventually, to evaluate the applicability and effectiveness of ROS promotion or ROS depletion strategies in cancer therapeutics. Special emphasis is laid on glial and glioma cells.

The manuscript is well structured and provides a throrough description of the model and the corresponding analyses. In particular the comprehensive appendix provides valueable information and completes the model specification. All in all the work appears sound and valid.

Therefore I only have a few minor comments.

- The validation of a mathmatical or computational model is a very critical step and should be performed very carefully and described in detail. In its current form this aspect comes a bit short. The authors should provide a separate paragraph and a more thorough description of their efforts in validating the entire model and/or, if done, distinct components of it. This should be complemented by a short discussion including statements under which conditions and for which cell types the authors consider their model as valid and predictive.

- In line with the previous aspect, the model has only been fitted against glial cells and glioma conditions - how do these results apply to other cancer types? Are the mechanisms described in the manuscript applicable to any other tumor type?

- The introduction is informative, but also a bit lengthy, and could be straightened at some points.

- The current way the parameter variation experiments of GTHP is described can cause some confusion: in table S1 the physiological range of the parameter Vm_[GTHP] is given as "(ranges: 0.2874 to 2.697 mM/hr)", which is in strong contrast to the parameter variation given in the manuscript (from 0.001mM/hr to 1.5 mM/hr). Here the authors should clearly communicate that (very) low expression of GTHP is characteristic for glioma cells, in particular because interesting changes in model behaviour are clearly below the normal conditions and occur up to 0.3mM/hr.

- Why has GTHO only been varied in combination with other parameters?

Finally, a nice have would be to have an endogenous mechanism causing the higher oxygen demand, instead of regulating the cellular oxygen demand by its affinity. This is fine for the current state of the model, but for future work such extension would be an interesting aspect.

**********

6. PLOS authors have the option to publish the peer review history of their article (what does this mean?). If published, this will include your full peer review and any attached files.

If you choose “no”, your identity will remain anonymous but your review may still be made public.

**Do you want your identity to be public for this peer review?** For information about this choice, including consent withdrawal, please see our Privacy Policy.

Reviewer #1: No

[NOTE: If reviewer comments were submitted as an attachment file, they will be attached to this email and accessible via the submission site. Please log into your account, locate the manuscript record, and check for the action link "View Attachments". If this link does not appear, there are no attachment files to be viewed.]

While revising your submission, please upload your figure files to the Preflight Analysis and Conversion Engine (PACE) digital diagnostic tool, https://pacev2.apexcovantage.com/. PACE helps ensure that figures meet PLOS requirements. To use PACE, you must first register as a user. Registration is free. Then, login and navigate to the UPLOAD tab, where you will find detailed instructions on how to use the tool. If you encounter any issues or have any questions when using PACE, please email us at figures@plos.org. Please note that Supporting Information files do not need this step.

# Author comment: Author response to Decision Letter 0

**DOI:** 10.1371/journal.pone.0235204.r003

20 May 2020

Response to Reviewer

To,

Prof. Salvatore V Pizzo

Academic Editor

PLOS ONE

Dear Prof. Pizzo,

First we would like to sincerely thank you for giving us the opportunity to perform the revision of our manuscript. We also thank the Reviewer for a detailed and critical review of our manuscript highlighting its deficiencies along with indicating the usefulness of our endeavour. The comments are insightful and have brought out several points that were not clearly stated in the earlier version of the paper. We are also thankful to the Reviewer for recognizing the importance of our work and effort, and advising us to submit the revised version of the manuscript. The manuscript is now thoroughly revised following the suggestions/comments made by the Reviewer (Highlighted in the revised Manuscript, Marked-up Copy) and the point to point answers are given below.

We hope this revised version will satisfy you and the reviewer and will be acceptable to the journal.

----------------------------------------------------------------------------------------------------

REPLY TO REVIEWERS’ COMMENTS

Reviewer #1: The manuscript by Bhowmick and Sarkar describes an ODE model of the coordinated function of several metabolic pathways involved in the regulation of the redox balance of the cell. The aim of the model is to study various ROS manipulation strategies in cancerous cells (compared to normal conditions), and, eventually, to evaluate the applicability and effectiveness of ROS promotion or ROS depletion strategies in cancer therapeutics. Special emphasis is laid on glial and glioma cells.

The manuscript is well structured and provides a thorough description of the model and the corresponding analyses. In particular the comprehensive appendix provides valuable information and completes the model specification. All in all, the work appears sound and valid.

Therefore I only have a few minor comments.

Reply: We thank the Reviewer for appreciating our efforts.

Comment 1: The validation of a mathematical or computational model is a very critical step and should be performed very carefully and described in detail. In its current form this aspect comes a bit short. The authors should provide a separate paragraph and a more thorough description of their efforts in validating the entire model and/or, if done, distinct components of it. This should be complemented by a short discussion including statements under which conditions and for which cell types the authors consider their model as valid and predictive.

Reply: Following the Reviewer's suggestion, a separate paragraph describing the validation of the model has been included in the manuscript. The earlier subsection ‘2.4. Parameter estimation and validation with experimental data’ has been split and a new subsection ‘2.5. Model Validation with experimental data’ under the Materials and Methods section has been added. A detailed description of model validation with proper references has been included in this subsection. Also, a short discussion on the conditions and cell types for which the model remains valid and predictive has been added by the end of this subsection. (Revised Manuscript, Line #243-295)

Comment 2: In line with the previous aspect, the model has only been fitted against glial cells and glioma conditions - how do these results apply to other cancer types? Are the mechanisms described in the manuscript applicable to any other tumor type?

Reply: As hypoxia and oxidative stress are characteristics of most tumors and antioxidant machinery remains central in maintaining redox balances in all cell types, the present model can be used to simulate various scenarios related to redox imbalances in other types of cancers as well. A note on the applicability of the model in other cancers has been included in the Discussion section (Manuscript, Line #761-769).

Comment 3: The introduction is informative, but also a bit lengthy, and could be straightened at some points.

Reply: As per the Reviewer’s suggestion, few of the details in the Introduction have been shortened and made straight. The changes can be tracked in the “Introduction” section of the ‘Revised Manuscript with Track Changes’ file.

Comment 4: The current way the parameter variation experiments of GTHP is described can cause some confusion: in table S1 the physiological range of the parameter Vm_[GTHP] is given as "(ranges: 0.2874 to 2.697 mM/hr)", which is in strong contrast to the parameter variation given in the manuscript (from 0.001mM/hr to 1.5 mM/hr). Here the authors should clearly communicate that (very) low expression of GTHP is characteristic for glioma cells, in particular because interesting changes in model behaviour are clearly below the normal conditions and occur up to 0.3mM/hr.

Reply: We thank the Reviewer for this useful comment. Through our simulation, we could indeed observe that a diminished expression of GTHP is a characteristic of gliomas. This has also been observed in other literatures, although the interpretations were made for brain tumors in general. This interpretation was missing in the earlier version of the manuscript and has been included now under the Results section (Manuscript, Line #396-404). 

Comment 5: Why has GTHO only been varied in combination with other parameters?

Response: For the analysis of cellular behavior under glioma scenario, we changed the parameter values of , , and as the system remained robust for most of the other parameter changes. We started with varying each of the parameters in combination with the other parameters, but could observe significant differences in h2o2 concentration only when glutathione reductase (GTHO) was varied in combination with other parameters. To understand this model property, we searched literature to check if varying expression of GTHO is an observable trait of gliomas. Zhu et. al. (J Neurochem. 2018; 144(1):93-10), in their study, they have shown that different GBM cell lines have different level of GTHO expression and overexpression of GTHO mediates drug resistance in these cells. Whereas, GTHO knockdown resensitize GTHO overexpressed cells to drug treatment. We corroborated this understanding with our model simulations to observe that varying GTHO significantly affect the thiol ratio of the gliomas along with significant difference in h2o2 concentration. This can be attributed to the biochemical property of the enzyme glutathione reductase which is to readily metabolize gssg to gsh through nadph-nadp+ mediated reaction so that gsh pool remains available for neutralizing oxidants maintaining a high gsh/gssg ratio. Thus, we propose that, at high expression of GTHO, the increased level of gsh/gssg ratio helps the cell to evade programmed cell death which would otherwise lead to apoptosis by the induction of toxicity due to uncontrolled increase in h2o2 levels. However, with lower expression of GTHO, a sharp decline in the gsh/gssg ratio drives the cell towards an apoptotic fate. A note on this has been added in the manuscript (Revised Manuscript, Line #560-567). 

Comment 6: Finally, it would be nice to have an endogenous mechanism causing the higher oxygen demand, instead of regulating the cellular oxygen demand by its affinity. This is fine for the current state of the model, but for future work such extension would be an interesting aspect.

Reply: We again thank the Reviewer for this suggestion. Endogenous factors like HIF1α expression, faulty Oxidative Phosphorylation, etc., in addition to the current state of the model will be considered in the future extension of this model. A note on this has been included in the Conclusion section (Revised Manuscript, Line #783-786).

We hope that the above replies will satisfy the reviewer and the manuscript will be acceptable for publication.

Attachment Submitted filename: Response to Reviewers.docx Click here for additional data file.

# Peer review: Decision Letter 1

**DOI:** 10.1371/journal.pone.0235204.r004

**Authors:** Salvatore V Pizzo

11 Jun 2020

Differential suitability of Reactive Oxygen Species and the Role of Glutathione in regulating paradoxical behavior in Gliomas: A Mathematical Perspective

PONE-D-19-27485R1

Dear Dr. Sarkar,

We’re pleased to inform you that your manuscript has been judged scientifically suitable for publication and will be formally accepted for publication once it meets all outstanding technical requirements.

Within one week, you’ll receive an e-mail detailing the required amendments. When these have been addressed, you’ll receive a formal acceptance letter and your manuscript will be scheduled for publication.

An invoice for payment will follow shortly after the formal acceptance. To ensure an efficient process, please log into Editorial Manager at http://www.editorialmanager.com/pone/, click the 'Update My Information' link at the top of the page, and double check that your user information is up-to-date. If you have any billing related questions, please contact our Author Billing department directly at authorbilling@plos.org.

If your institution or institutions have a press office, please notify them about your upcoming paper to help maximize its impact. If they’ll be preparing press materials, please inform our press team as soon as possible -- no later than 48 hours after receiving the formal acceptance. Your manuscript will remain under strict press embargo until 2 pm Eastern Time on the date of publication. For more information, please contact onepress@plos.org.

Kind regards,

Salvatore V Pizzo

Academic Editor

PLOS ONE

Additional Editor Comments (optional):

Reviewers' comments:

Reviewer's Responses to Questions

**Comments to the Author**

1. If the authors have adequately addressed your comments raised in a previous round of review and you feel that this manuscript is now acceptable for publication, you may indicate that here to bypass the “Comments to the Author” section, enter your conflict of interest statement in the “Confidential to Editor” section, and submit your "Accept" recommendation.

Reviewer #1: All comments have been addressed

**********

2. Is the manuscript technically sound, and do the data support the conclusions?

The manuscript must describe a technically sound piece of scientific research with data that supports the conclusions. Experiments must have been conducted rigorously, with appropriate controls, replication, and sample sizes. The conclusions must be drawn appropriately based on the data presented. 

Reviewer #1: Yes

**********

3. Has the statistical analysis been performed appropriately and rigorously? 

Reviewer #1: N/A

**********

4. Have the authors made all data underlying the findings in their manuscript fully available?

The PLOS Data policy requires authors to make all data underlying the findings described in their manuscript fully available without restriction, with rare exception (please refer to the Data Availability Statement in the manuscript PDF file). The data should be provided as part of the manuscript or its supporting information, or deposited to a public repository. For example, in addition to summary statistics, the data points behind means, medians and variance measures should be available. If there are restrictions on publicly sharing data—e.g. participant privacy or use of data from a third party—those must be specified.

Reviewer #1: Yes

**********

5. Is the manuscript presented in an intelligible fashion and written in standard English?

PLOS ONE does not copyedit accepted manuscripts, so the language in submitted articles must be clear, correct, and unambiguous. Any typographical or grammatical errors should be corrected at revision, so please note any specific errors here.

Reviewer #1: Yes

**********

6. Review Comments to the Author

Please use the space provided to explain your answers to the questions above. You may also include additional comments for the author, including concerns about dual publication, research ethics, or publication ethics. (Please upload your review as an attachment if it exceeds 20,000 characters)

Reviewer #1: (No Response)

**********

7. PLOS authors have the option to publish the peer review history of their article (what does this mean?). If published, this will include your full peer review and any attached files.

If you choose “no”, your identity will remain anonymous but your review may still be made public.

**Do you want your identity to be public for this peer review?** For information about this choice, including consent withdrawal, please see our Privacy Policy.

Reviewer #1: No

# Editor report: Acceptance letter

**DOI:** 10.1371/journal.pone.0235204.r005

**Authors:** Salvatore V Pizzo

15 Jun 2020

PONE-D-19-27485R1 

Differential suitability of Reactive Oxygen Species and the Role of Glutathione in regulating paradoxical behavior in Gliomas: A Mathematical Perspective 

Dear Dr. Sarkar:

I'm pleased to inform you that your manuscript has been deemed suitable for publication in PLOS ONE. Congratulations! Your manuscript is now with our production department. 

If your institution or institutions have a press office, please let them know about your upcoming paper now to help maximize its impact. If they'll be preparing press materials, please inform our press team within the next 48 hours. Your manuscript will remain under strict press embargo until 2 pm Eastern Time on the date of publication. For more information please contact onepress@plos.org.

If we can help with anything else, please email us at plosone@plos.org. 

Thank you for submitting your work to PLOS ONE and supporting open access. 

Kind regards, 

PLOS ONE Editorial Office Staff

on behalf of

Dr. Salvatore V Pizzo 

Academic Editor

PLOS ONE
